# Profiling of ERBB receptors and downstream pathways reveals selectivity and hidden properties of ERBB4 antagonists

**DOI:** 10.1016/j.isci.2024.108839

**Published:** 2024-01-09

**Authors:** Lukša Popović, Jan P. Wintgens, Yuxin Wu, Ben Brankatschk, Sascha Menninger, Carsten Degenhart, Niels Jensen, Sven P. Wichert, Bert Klebl, Moritz J. Rossner, Michael C. Wehr

**Affiliations:** 1 Research Group Cell Signalling, Department of Psychiatry and Psychotherapy, LMU University Hospital, LMU Munich, Nussbaumstrasse 7, 80336 Munich, Germany; 2 Systasy Bioscience GmbH, Balanstrasse 6, 81669 Munich, Germany; 3 Lead Discovery Center GmbH, Otto-Hahn-Strasse 15, 44227 Dortmund, Germany; 4 Section of Molecular Neurobiology, Department of Psychiatry and Psychotherapy, LMU University Hospital, LMU Munich, Nussbaumstrasse 7, 80336 Munich, Germany

**Keywords:** Pharmacology, Natural sciences, Biological sciences, Biochemistry, Cell biology

## Abstract

ERBB receptor tyrosine kinases are involved in development and diseases like cancer, cardiovascular, neurodevelopmental, and mental disorders. Although existing drugs target ERBB receptors, the next generation of drugs requires enhanced selectivity and understanding of physiological pathway responses to improve efficiency and reduce side effects. To address this, we developed a multilevel barcoded reporter profiling assay, termed ‘ERBBprofiler’, in living cells to monitor the activity of all ERBB targets and key physiological pathways simultaneously. This assay helps differentiate on-target therapeutic effects from off-target and off-pathway side effects of ERBB antagonists. To challenge the assay, eight established ERBB antagonists were profiled. Known effects were confirmed, and previously uncharacterized properties were discovered, such as pyrotinib’s preference for ERBB4 over EGFR. Additionally, two lead compounds selectively targeting ERBB4 were profiled, showing promise for clinical trials. Taken together, this multiparametric profiling approach can guide early-stage drug development and lead to improved future therapeutic interventions.

## Highlights

• Multilevel barcoded reporter assay for profiling of ERBB receptors and pathways
• Discriminating on-target from off-target effects of ERBB receptor antagonists
• Profiled eight known ERBB receptor antagonists and two new ERBB4 selective leads
• Pyrotinib and novel compound B show a substantial preference for ERBB4 over EGFR

## Graphical abstract

**Figure:**
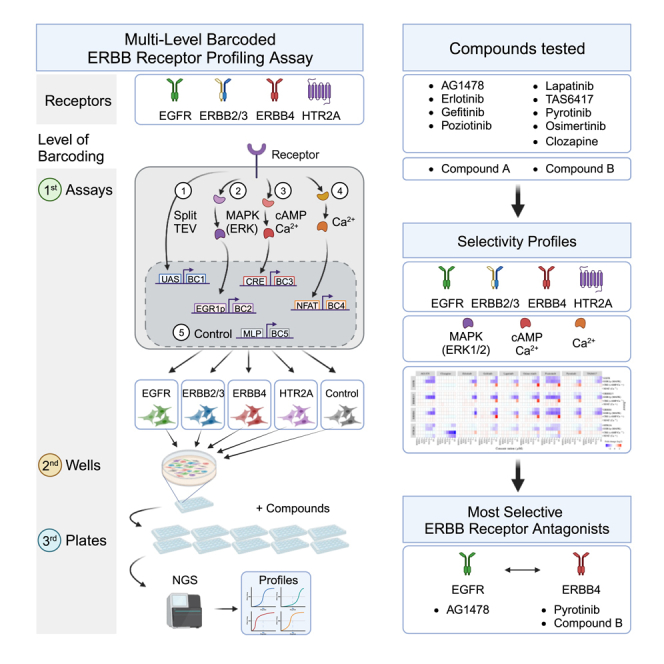

## Introduction

ERBB receptor family kinases are a sub family of receptor tyrosine kinases (RTKs) and are implicated in various human disorders, such as various types of cancer, e.g., breast cancer and non-small cell lung cancer, as well as neurological disorders and mental disorders, including schizophrenia.^1^^,^^2^^,^^3^^,^^4^ The ERBB family consists of four type I transmembrane receptors, which are the epidermal growth factor receptor (EGFR, also known as ERBB1), ERBB2 receptor tyrosine kinase 2 (also known as HER2), ERBB3 (HER3), and ERBB4 (HER4).^2^ ERBB receptors are activated by epidermal growth factor (EGF) and heregulin-like family ligands and can homo- and heterodimerize, leading to the phosphorylation on cytoplasmic tyrosine residues and the activation of cellular signaling cascades. While ERBB2 does not bind any endogenous ligand, it can still signal downstream when dimerized.^5^^,^^6^^,^^7^ ERBB3 favors heterodimerization with its preferred dimerization partner ERBB2 due to an impaired kinase domain to elicit cellular signaling.^8^ Canonical cellular signaling pathways initiated by activated ERBB receptors are the KRAS-RAF-mitogen-activated protein kinase (MAPK) cascade, phosphatidylinositol 3-kinase (PI3-K), and STAT signaling.^9^^,^^10^ In particular, the MAPK axis, which is initiated by the binding of the growth factor receptor-bound protein 2 (GRB2) adapter to phosphorylated tyrosine residues of ERBB receptors and ultimately results in extracellular regulated kinase 1 and 2 (ERK1/2) activation, is a bona-fide pathway that links ERBB receptor activity to cellular phenotypes, like cell cycle progression and proliferation.^11^^,^^12^

Cancer formation is linked to an increased expression of EGFR,^13^ ERBB2,^14^ ERBB3,^15^ and ERBB4.^16^^,^^17^ Notably, ERBB receptors play overlapping roles in various cancers, with, however, distinct mechanisms.^18^ For example, both EGFR and ERBB4 are linked to colorectal cancer^19^^,^^20^ and non-small cell lung cancer (NSCLC),^21^ but mutations for either EGFR or ERBB4 generally occur separately. Inhibiting ERBB signaling using small molecules is an attractive approach to treat multiple types of cancer. For example, gefitinib, the first EGFR tyrosine kinase inhibitor (TKI) developed, was approved by the US Food and Drug Association (FDA) in 2003 to treat NSCLC,^22^ followed by the FDA approval of erlotinib for NSCLC in 2004^23^ and lapatinib for the treatment of ERBB2 positive breast cancer in 2007.^24^ However, resistance patterns to ERBB inhibitor treatment emerged due to secondary mutations at gatekeeper residues (e.g., T790M in EGFR) i.e., larger residues sterically impeded inhibitor binding.^25^ Furthermore, triggering alternative downstream signaling routes and the activation of bypass survival tracks via other RTKs also accounts for the acquired drug resistance to ERBB receptors.^26^^,^^27^ To avoid resistance, various third generation TKI drugs were developed, such as osimertinib targeting the sensitizing mutation EGFR-T790M to treat NSCLC.^28^^,^^29^ Furthermore, overactive ERBB4 is associated with reduced interneuron activity in the prefrontal cortex leading to an imbalance of excitation and inhibition, and consecutively to symptoms of schizophrenia.^30^^,^^31^ Thus, ERBB4 is regarded as a critical target for schizophrenia, and drug development is required to identify compounds that selectively target ERBB4,^32^ but not EGFR, its most related receptor within the ERBB family.

As activated ERBB receptors can form homo or heterodimers restricted to their sub family,^2^ targeting only one family member and modulating its downstream signaling is difficult. In addition to those challenges, the development of kinase inhibitors is challenging in general, as kinases are targeted by only 3% of all marketed drugs.^33^ Notably, kinases and especially RTKs, together with G protein-coupled receptors (GPCRs), represent one of the two most important drug targets in human cells.^33^ Biochemical binding assays and FRET-based assays are frequently used to profile compounds that interact with RTKs, including ERBB receptors, and offer valuable insights into the binding properties of various compounds. However, it is essential to recognize their limitations, particularly the lack of physiological context in test tube (*in-vitro*) settings, the inability to account for physiologically relevant interactions at the cell membrane (which is crucial for RTKs), and the potential artifacts from those *in-vitro* conditions.^34^ Standard reporter gene assays in living cells may solve some aspects of the limitations mentioned above. However, single assays are not able to deliver functionally diverse phenotypes from the very same cell population (i.e., an agonistic and antagonistic effect monitored on different pathways from one well). Therefore, the development of a holistic cell-based assay system that enables the monitoring of ERBB activities and downstream pathways may substantially contribute to the advancement of better therapeutic agents targeting ERBB receptors.

Here, we describe a barcoded profiling assay, termed ‘ERBBprofiler’, to simultaneously assess the activity of ERBB receptors in their native environment at the cell membrane and their downstream responses to key cellular pathways in living cells. Selective activities of individual ERBB receptors and pathway responses associated with receptor activation or inhibition can be monitored in the same well upon drug treatment. While ERRB receptor activities at the membrane were monitored by split TEV recruitment assays, pathway responses were measured by optimized nuclear pathway sensors. By profiling target selectivity and physiological responses of established drugs and compounds that are either approved, currently tested in clinical phases, or failed, we have demonstrated the capabilities of the cell-based assay platform to identify relevant features of ERBB antagonists. Lastly, we used the assay platform to profile novel ERBB4 selective antagonists, supporting the notion that the profiling assay can be used to accelerate early drug discovery campaigns.

## Results

### Design of a barcoded ERBB family receptor and pathway profiling assay

**Figure 1:**
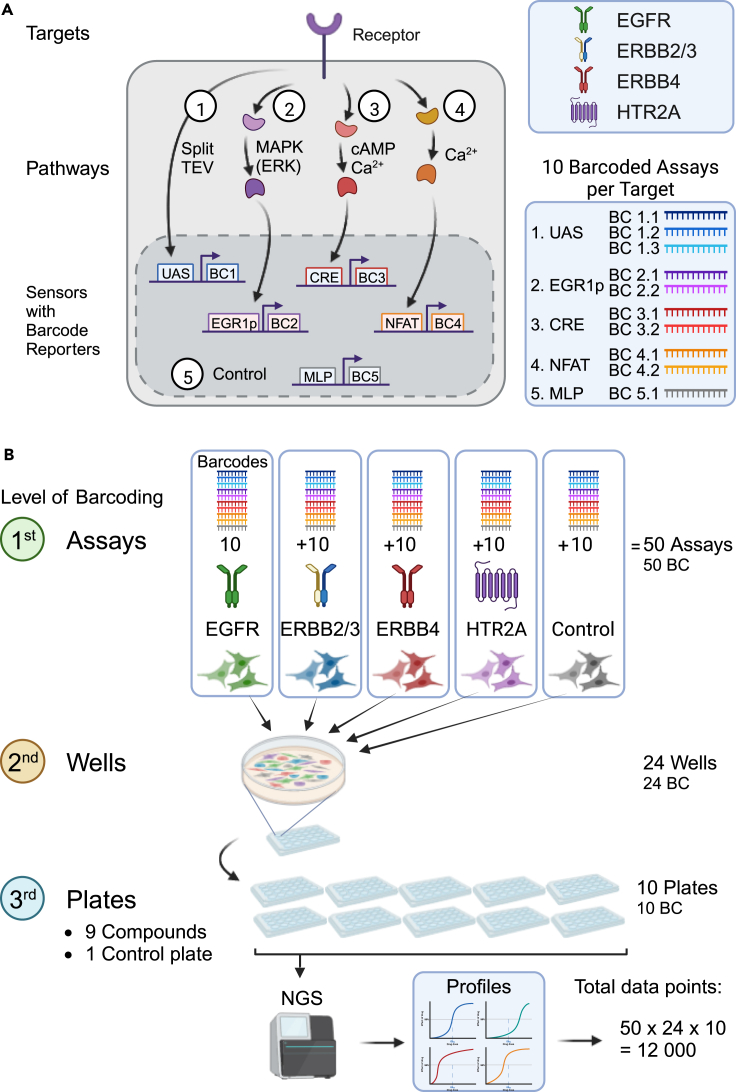
Design of a fully integrated, multiplexed assay to profile activities of ERBB receptor family and physiological signaling in parallel (A) Summary schematic showing target and pathway assays of the barcoded ERBBprofiler assay. Activities of receptors were monitored using split TEV assays, while activities of pathways were measured using pathway sensors. BC, barcode. Upper inset: targets used in this study. Lower inset: Barcode reporters for targets (UAS), pathways (EGR1p for MAPK/ERK signaling, CRE for cAMP/Ca^2+^ signaling, and NFAT for Ca^2+^ signaling), and a control (MLP). (B) Workflow for molecular barcoding of assays, wells, and plates. On the first level of barcoding, 50 assays, each with a unique barcode, are monitored in one well. On the second level, the lysates of the wells are barcoded, and on the third level, the plates are barcoded. Barcodes are sequenced using next generation sequencing (NGS), and data are analyzed. In a typical experiment comprising ten 24-well plates, 12,000 data points are generated from ten Tag&Pool (a method to process 24 lysates combined, see STAR Methods section) samples. See also Figure S1 and Table S1.

Profiling of both ERBB targets and downstream pathway activities was performed by genetically encoded split TEV and pathway reporter gene assays, respectively, and requires a readout that is amenable to parallelization (Figure 1A). Therefore, the firefly luciferase reporter of each reporter plasmid was replaced by a unique DNA barcode that is transcribed into an RNA barcode, which can be isolated from cells and quantified by next-generation sequencing (NGS).^35^^,^^36^ For establishing the multiparametric ERBB receptor profiling assay, we first cloned small (n ≤ 20) libraries of the reporter plasmids encoding (1) a barcoded Gal4-VP16 reporter for split TEV assays consisting of clustered upstream activating sequences (10xUAS), (2) a barcoded pathway sensor for MAP kinase signaling based on the early growth response 1 promotor (EGR1p sensor), (3) a barcoded pathway sensor for cAMP/calcium signaling based on cAMP response element (CRE sensor), (4) a barcoded pathway sensor for calcium signaling based on the response element of the nuclear factor of activated T-cells (NFAT sensor), and (5) a barcoded sensor for assessing baseline activities based on the major late promoter (MLP) from adenovirus. For the simultaneous monitoring of target and pathway activities, a subset of these barcode reporters (3 unique barcodes for UAS, 2 unique barcodes each for CRE, EGR1p, NFAT, and 1 for MLP) was transfected with plasmids encoding split TEV compatible ERBB receptors (i.e., EGFR, ERBB2 in combination with ERBB3, or ERBB4) and the split TEV RTK adapter 3xSH2-GRB2^37^ into PC12 cells for ERBB receptor assays. In addition, we wished to add an unrelated target as a control assay. Thus, another subset of the reporter plasmids was transfected with the split TEV compatible serotonin receptor 2A (HTR2A), a Gα_q_-coupled GPCR, and the split TEV GPCR adapter ARBB2-CTEV^38^ for an HTR2A assay (Figures S1A–S1F; Table S1). Therefore, each batch of cells contained the plasmids encoding a receptor, its cognate adapter, and the split TEV and pathway reporters with a set of unique barcodes enabling to assess the activities of one target, i.e., an ERBB receptor, or HTR2A, and its downstream signals. Multiplexing of target and pathway assays was then further conducted by mixing cell batches into one well and continued at the plate level. Thus, barcoding occurred at the levels of single assays, wells, and plates, and enabled the generation of thousands of data points from one ERBBprofiler experiment (Figure 1B). In a typical profiling experiment, batches of transfected PC12 cells were mixed, plated onto 24-well plates, cultured for 20 h, starved for 16 h in medium with reduced serum conditions, and treated with increasing doses of epidermal growth factor (EGF, ligand for EGFR), EGF-like domain (EGFld, ligand for ERBB3 and ERBB4), and serotonin (ligand for HTR2A) for 6 h before lysis (Figure S1G). To proceed to the sequencing of barcodes using next-generation sequencing and to reduce handling, a unique DNA barcode oligo, termed well barcode, was added to each lysate originating from one well, enabling the tracking of individual lysates in a pool. Next, all lysates from one 24-well plate were pooled for combined processing, a process called ‘Tag&Pool’ (read ‘Tag-and-Pool’, see STAR Methods) (Figures S1H and S1I).

### Selectivity of ERBB receptor activation, HTR2A activation, and their downstream signaling by single ligands correlate in barcoded assays

**Figure 2:**
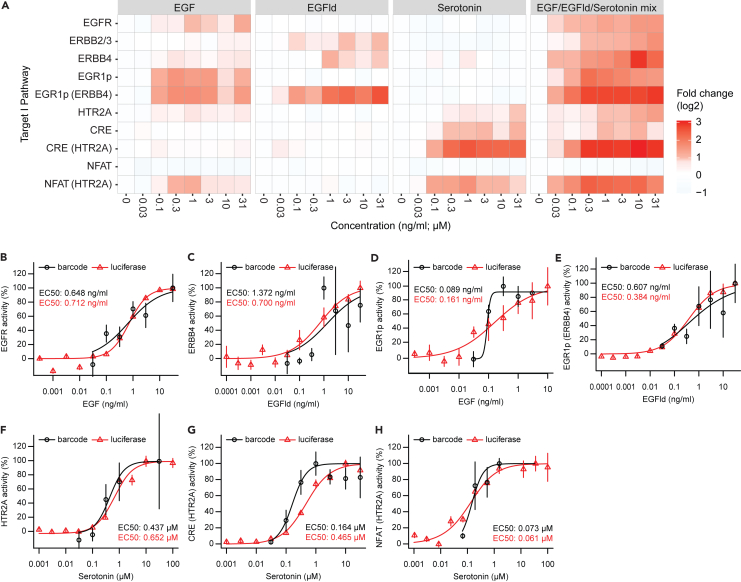
Selectivity of receptor activation and downstream signaling by single ligands correlate in barcoded assays (A) Heatmap showing stimulation profiles on ERBB receptors, HTR2A, and downstream signaling pathways. Assays for receptors were performed using barcoded split TEV, assays for signaling pathways with pathway sensors coupled to barcodes. Compound effects are shown as log2-transformed fold change. (B–H) Barcoded assays align with luciferase readouts. Visualization of selected data from (A), comparing barcoded assays (black) with luciferase assay readouts (red). Assays for receptors were performed using split TEV, assays for signaling pathways with pathway sensors. Dose response graphs for EGFR (B), ERBB4 (B), EGR1p only (D), and EGR1p and ERBB4 transfected (E), HTR2A (F), CRE and HTR2A transfected (G), and NFAT and HTR2A transfected (H) with single stimuli applied at increasing concentrations. EGFld, EGF-like domain. Error bars represent SEM, n = 3 for barcoded assays, and n = 6 for luciferase assays. See also Figure S2 and Table S2.

In the initial ERBBprofiler assay experiment, we examined the impact of EGF, EGFld, and serotonin on the activation on ERBB receptors, HTR2A, and their key downstream pathways. While EGF treatment selectively stimulated the activity of EGFR, addition of EGFld led to the activation of ERBB2/3 and ERBB4 (Figure 2A). The responsiveness to the ligands was clearly observed in dose-response graphs for EGFR (Figure 2B) and ERBB4 (Figure 2C). PC12 endogenously express EGFR, and EGFR stimulation leads to the activation of the ERK1/2 branch of MAPK signaling.^39^ In our cellular profiling assay, this branch of MAPK signaling was captured by the EGR1p sensor. As expected, PC12 cells that were transfected with the EGR1p sensor only responded to increasing EGF concentrations (Figures 2A and 2D). Conversely, EGFld treatment did not lead to any response. When ERBB4 was co-transfected with the EGR1p sensor, EGFld indeed induced an EGR1p response, as evident from an increase in EGR1p sensor activity (Figures 2A and 2E). Serotonin treatment selectively activated HTR2A, and physiologically linked calcium signaling was induced when HTR2A was activated, as indicated by increased CRE and NFAT reporter activities (Figures 2A and 2F–2H). EGF treatment led to a low activation of the NFAT sensor in the presence of co-transfected HTR2A, a finding that can be explained by crosstalk between EGFR and HTR2A.^40^ Notably, neither EGF nor EGFld activated CRE and NFAT sensors in the absence of HTR2A, nor did serotonin lead to the activation of the EGR1p sensor, supporting the notion that the stimuli were selective for both targets and physiological pathways. To benchmark the performance of barcoded assays, target and pathway assays for EGFR, ERBB4, and HTR2A were replicated with a luciferase-based readout at dose response (Figures 2B–2H). Barcoded and luciferase assays showed comparable dose-response curves and EC_50_ values for each assay tested. Responses to target activation (measured by split TEV) and cellular signaling (measured by pathway sensors) correlated for both barcoded assays and luciferase assays (see Figure S2). In addition, cells were treated with a mix of all three ligands to assess any potential synergistic effects among ligands, as this mix of ligands was planned to be applied in antagonist profiling assays to reduce sample numbers. Notably, the effects observed by the individual stimuli and the mix matched for both targets and pathway assays (Figures 2A and S2), enabling a simultaneous assessment in compound profiling assays. All EC_50_ values for barcoded assays are summarized in Table S2.

### The ERBBprofiler assay reveals known and previously uncharacterized selectivity properties of ERBB receptor antagonists

**Figure 3:**
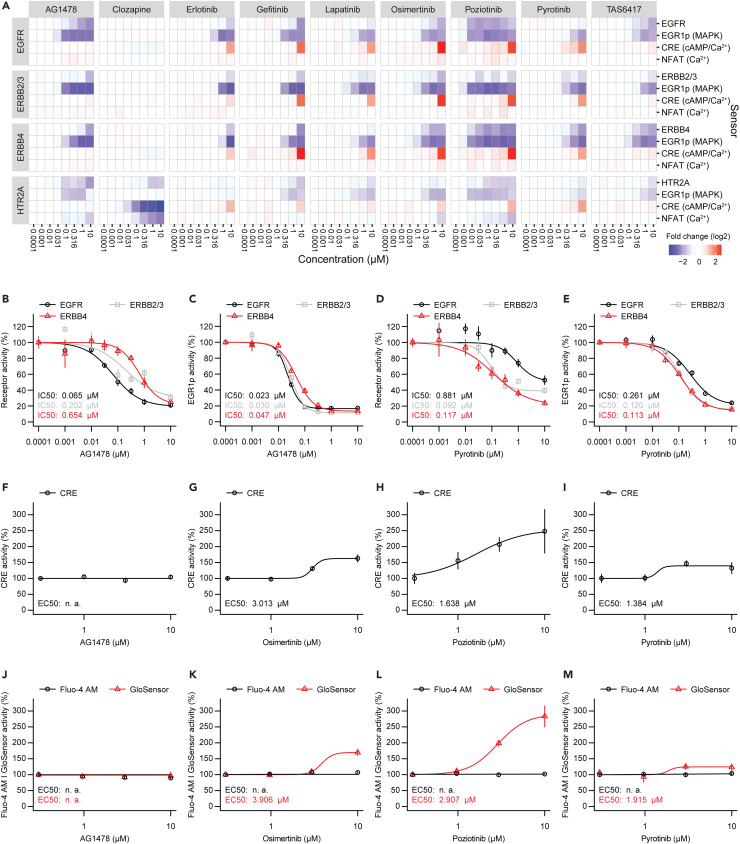
The barcoded ERBBprofiler reveals known and previously uncharacterized selectivity properties of ERBB receptor antagonists (A) Heatmap showing antagonistic effects of compounds on ERBB receptors, HTR2A, and downstream signaling pathways in PC12 cells. Assays for receptors were performed using barcoded split TEV assays, assays for signaling pathways with barcoded pathway sensors. In addition to the increasing concentrations of the compounds shown, all assays contained constant concentrations of EGF (30 ng/mL), EGF-like domain (10 ng/mL), and serotonin (1 μM). Compound effects are shown as log2-transformed fold change. (B–E) Dose response graphs comparing drug selectivity for receptors EGFR and ERBB4 (B, D) and downstream MAPK signaling (C, E) of compounds AG1478 (B, C), and pyrotinib (D, E). Data was extracted from the heatmap shown in (A). n = 3. (F–I) Dose response graphs for CRE sensor responses in PC12 cells using luciferase as readout for AG1478 (F), osimertinib (G), poziotinib (H), and pyrotinib (I). In addition to the increasing concentrations of the compounds shown, all assays contained the constant stimulation mix as in (A). (J–M) Dose response graphs for calcium and cAMP assays using Fluo-4 a.m. and GloSensor, respectively, as readouts in PC12 cells treated with increasing concentrations of AG1478 (J), osimertinib (K), poziotinib (L), and pyrotinib (M). As in luciferase assays, the constant stimulation mix was constantly present, next to the mentioned compounds. Error bars represent SEM, n = 3 for barcode assays (B-E), n = 6 for luciferase, Fluo-4 a.m., and GloSensor assays (F-M). See also Figure S3, Tables S3 and S4.

Next, we sought to challenge the barcoded ERBBprofiler assay and selected various ERBB receptor antagonists, which included approved drugs (erlotinib, gefitinib, lapatinib, osimertinib),^22^^,^^24^^,^^41^^,^^42^ but also a failed compound (AG1478),^43^ and drug candidates that are in a clinical trial stage (poziotinib, pyrotinib, TAS6417).^44^^,^^45^^,^^46^ Clozapine was selected as an approved drug for targeting HTR2A.^47^ As antagonist assays require activated receptors and pathways, we selected the mix of ligands to stimulate ERBB receptors and HTR2A, as well as signaling pathways. As receptors and pathway sensors were sufficiently activated at a concentration of 30 ng/mL EGF, 10 ng/mL EGFld, and 1 μM serotonin (Figure 2A), dose response treatments for all selected drugs were performed in PC12 cells using those agonist concentrations and the same experimental setup as outlined before. Agonists were applied as a mix to reduce sample number. To further simplify handling, antagonist and agonist treatments were conducted simultaneously (Figure S1G). From a total of 240 samples, batches of 24 samples were pooled at the lysis stage using the Tag-and-Pool process to produce ten samples that were processed for barcode sequencing, yielding 12000 data points in total (Figure 1B). For each compound, we obtained a dose-response dataset for each receptor (EGFR, ERBB2/3, ERBB4, and HTR2A) measured by split TEV assays and pathway sensors (EGR1p for the ERK branch of MAPK signaling, CRE for cAMP/calcium signaling, NFAT for calcium signaling) linked to each receptor (Figures 1A and 3A).

All ERBB antagonists inhibited activities of stimulated EGFR, ERBB2/3, and ERBB4, indicating a pan-ERBB-selective profile, albeit with different selectivity (see Table S3 for all IC_50_ values). Erlotinib and poziotinib had similar affinities for all ERBB receptors, and rather displayed an evenly distributed pan-ERBB selectivity profile (Figures 3A and S3). Gefitinib showed a mild preference for EGFR (EGFR IC_50_, 0.51 μM; ERBB2/3 IC_50_, 0.752 μM; ERBB4 IC_50_, 1.23 μM) (Figure S3). Conversely, lapatinib had a preference for ERBB2/3 over EGFR and ERBB4 (EGFR IC_50_, 0.942 μM; ERBB2/3 IC_50_, 0.093 μM; ERBB4 IC_50_, 0.825 μM), which is consistent with previous observations we made in standard split TEV luciferase assays.^37^ Similarly, osimertinib favorably antagonized ERBB2/3 and ERBB4 over EGFR (EGFR IC_50_, 0.201 μM; ERBB2/3 IC_50_, 0.032 μM; ERBB4 IC_50_, 0.082 μM). TAS6417 preferentially inhibited both EGFR and ERBB4 over ERBB2/3, although these differences were minor (EGFR IC_50_, 0.092 μM; ERBB2/3 IC_50_, 0.206 μM; ERBB4 IC_50_, 0.091 μM). In agreement with published data from *in-vitro* assays,^48^ our data confirmed antagonistic effects of TAS6417 on ERBB2/3 and ERBB4 activity for the first time *in cellulo*, as well as on ERK signaling downstream of ERBB2/3 and ERBB4. Clozapine, chosen as non-ERBB receptor inhibitor control, selectively antagonized HTR2A and downstream pathways as measured by CRE and NFAT sensors, but did not inhibit any ERBB receptor, nor ERBB mediated signaling (Figures 3A and S3). However, the most striking differences in antagonizing EGFR or ERBB4 for the selected compounds were identified for AG1478 and pyrotinib. AG1478 showed a clear preference for antagonizing EGFR over ERBB2/3 and ERBB4 activities in barcoded split TEV assays (EGFR IC_50_, 0.065 μM; ERBB2/3 IC_50_, 0.202 μM; ERBB4 IC_50_, 0.654 μM) (Figure 3B). Conversely, pyrotinib preferentially antagonized ERBB4 activity over EGFR activity in our assay (EGFR IC_50_, 0.881 μM; ERBB4 IC_50_, 0.117 μM) (Figure 3C). Notably, pyrotinib is reported to efficiently bind to EGFR in biochemical assays,^49^ but activity on ERBB4 has not been reported so far.^50^ In the pathway based EGR1p readout, the preferential antagonistic effects of AG1478 and pyrotinib were still measurable, but less pronounced (Figures 3D and 3E). All measured effects for the eight ERBB antagonists and clozapine are summarized in Table S3 and compared to known effects from literature in Table S4.

When comparing our results to IC_50_ values of phospho-blots and cell proliferation studies, we either had comparable potency values (e.g., for AG1478 on EGFR,^51^ erlotinib on phospho-ERK,^52^ osimertinib on ERBB4,^28^ poziotinib on EGFR and ERBB2/3,^53^ and TAS6417 on EGFR^48^) or an order of magnitude higher IC_50_ values, possibly due to the use of different cell lines and assay readouts (e.g., for erlotinib on EGFR, gefitinib on EGFR and ERBB4,^54^^,^^55^ osimertinib on EGFR,^56^ and pyrotinib on EGFR.^49^ Furthermore, our data were comparable to results for clozapine effects with three other assays, namely the commercial DiscoveRx PathHunter assay for the HTR2A receptor, a cAMP accumulation assay, and a Fluo-4 assay to measure Ca^2+^.^57^^,^^58^^,^^59^

Of note, many ERBB antagonists, including osimertinib, poziotinib, and pyrotinib, caused an upregulation of the CRE sensor at high compound concentration of 10 μM. An increased CRE sensor activity may pinpoint to both increased cAMP and calcium concentrations that may arise from cellular stress.^60^ To validate the results from the barcoded profiling assay, we conducted single luciferase assays using the CRE sensor as readout for these three compounds and AG1478, as the latter did not cause any increased CRE sensor activities in the barcoded assay. In PC12 cells that were transfected with the CRE sensor only, we found similar patterns of CRE sensor activation at high compound concentrations of osimertinib, poziotinib, and pyrotinib, but not for AG1478 (Figures 3F–3I), suggesting a cell intrinsic response caused by a subset of inhibitors. To identify whether the CRE sensor responded to increased cAMP or calcium concentrations, we conducted orthogonal assays in PC12 cells to measure cAMP using a GloSensor assay, while calcium was assessed using Fluo-4 a.m., a cell permeable calcium indicator. AG1478 treatment did not cause any increase in neither cAMP nor calcium (Figure 3J), while higher concentrations (≥3 μM) of osimertinib (Figure 3K), poziotinib (Figure 3L), and pyrotinib (Figure 3M) resulted in elevated cAMP, but not calcium levels.

### Pyrotinib reveals selectivity for ERBB4 over EGFR

**Figure 4:**
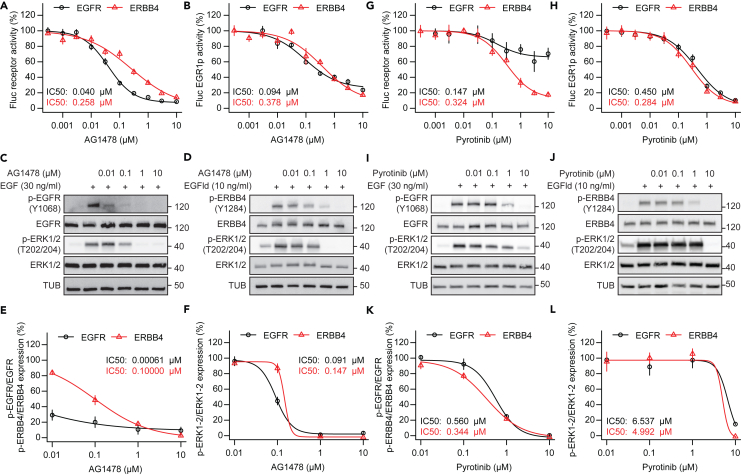
Pyrotinib reveals selectivity for ERBB4 over EGFR (A and B) Dose response assays comparing AG1478 selectivity for receptors EGFR and ERBB4 (A) and downstream MAPK signaling (B) using firefly luciferase assays in PC12 cells. Assays for receptors were performed using split TEV, assays for MAPK signaling were conducted with an EGR1p pathway sensor. In addition to the increasing concentrations of AG1478, EGFR and ERBB4 assays contained a constant concentration of EGF (30 ng/mL) or EGF-like domain (10 ng/mL), respectively. (C and D) Western blot analyses of *p*-EGFR (in A549 cells) (C) and *p*-ERBB4 (in T-47 cells) (D) using increasing concentrations of AG1478. (E and F) Quantification of (C) and (D). (G and H) Dose response assays comparing pyrotinib selectivity for receptors EGFR and ERBB4 (G) and downstream MAPK signaling (H) using firefly luciferase assays in PC12 cells. Assays were conducted as in (A, B). (I and J) Western blot analyses of *p*-EGFR (in A549 cells) (I) and *p*-ERBB4 (in T-47 cells) (J) using increasing concentrations of pyrotinib. (K and L) Quantification of (I) and (J). Error bars represent SEM, n = 6 for luciferase assays (A, B, G, H), n = 3 for Western blot assays (E, F, K, L).

A key feature of the established ERBB profiling assay should be that it can discriminate between EGFR and ERBB4 selective compounds. Next, we aimed to validate the barcode assay results for AG1478 and pyrotinib using single luciferase assays and Western blotting as orthogonal assays. AG1478 inhibited EGFR over ERBB4 in split TEV luciferase assays (Figure 4A). However, this preference for EGFR was not present anymore when the EGR1p pathway sensor was used as readout (Figure 4B), indicating that a direct coupling of the reporter to the target, as in split TEV assays, is required to detect subtle differences. To confirm AG1478’s activity on EGFR over ERBB4, A549 cells, which endogenously express EGFR,^61^ and T-47D cells, which endogenously express ERBB4,^37^^,^^62^ were treated with constant concentrations of agonists (i.e., EGF for A549 cells, and EGFld for T-47D cells) and increasing concentrations of AG1478. Indeed, AG1478 preferentially antagonized phospho-EGFR over phospho-ERBB4 in Western blot analyses (Figures 4C–4F). In addition, AG1478 inhibited EGFR mediated downstream ERK signaling, a finding that was quantified *in cellulo* for the first time by our barcoded assay (Figures 3A–3C). These results were validated by the luciferase assay using the EGR1p sensor as well as biochemically by assessing phospho-ERK1/2 in a Western blot analysis (Figures 4B and 4F). In sum, the preference for AG1478-mediated EGFR over ERBB4 inhibition was present at receptor level but was lost at pathway level in both the barcoded assays and Western blot assays.

Conversely, pyrotinib preferentially inhibited ERBB4 activity over EGFR activity in split TEV receptor assays (Figure 4G). However, pyrotinib’s preference for ERBB4 was compromised in pathway assays using the EGR1p sensor as downstream readout (Figure 4H). To confirm pyrotinib’s activity on ERBB4 over EGFR, we treated A549 cells and T-47D cells with constant concentrations of agonists (i.e., EGF for A549 cells, and EGFld for T-47D cells) and increasing concentrations of pyrotinib. Indeed, pyrotinib treatment led to a more potent inhibition of ERBB4 activity as measured by phospho-ERBB4 and phospho-EGFR (Figures 4I–4L). Similar to the barcoded and luciferase EGR1p pathway assays, EGFR and ERBB4-dependent phospho-ERK1/2 signals did not substantially differ in pyrotinib treated cells (Figures 4H and 4L). Taken together, the barcoded ERBB profiling assay can assess antagonistic actions on ERBB receptors and key physiological pathways, assess a compound’s selectivity, and identify potential side effects in one experiment.

### Discovery of novel ERBB4 selective antagonists

Previously, we identified spironolactone as ERBB4 antagonist in a drug repurposing screen, while spironolactone’s metabolite canrenone did not inhibit ERBB4 activity.^32^ To test the capabilities of the barcoded ERBBprofiler assay, we treated PC12 assay cells with increasing concentrations of both spironolactone and canrenone and agonists. Results confirmed the antagonistic effect of spironolactone on ERBB4 with similar activity (IC_50_: 4.327 μM) (Figure S4). Conversely, canrenone did not inhibit any ERBB receptor nor pathway monitored. Furthermore, we found that spironolactone inhibited HTR2A at 10 μM, but not at lower concentrations (Figure S4).

**Figure 5:**
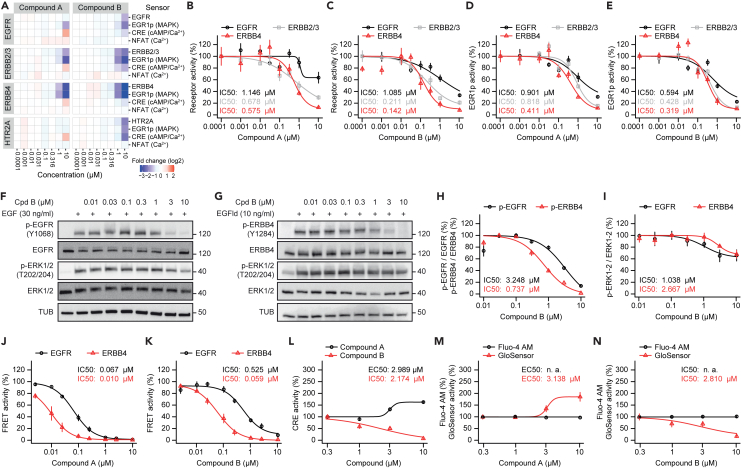
The barcoded ERBBprofiler reveals novel ERBB4 selective antagonists (A) Heatmap showing antagonistic effects of LDC compounds on ERBB receptors, HTR2A, and downstream signaling pathways in PC12 cells. Assays for receptors were performed using barcoded split TEV assays, assays for signaling pathways using barcoded pathway sensors. In addition to the increasing concentrations of the compounds shown, all assays contained constant concentrations of EGF (30 ng/mL), EGF-like domain (10 ng/mL), and serotonin (1 μM). (B–E) Dose response graphs comparing drug selectivity for ERBB4 over EGFR (measured with split TEV) (B, C) and downstream MAPK signaling (measured with the EGR1p sensor) (D, E) of compound A (B, D), and compound B (C, E). Data was extracted from the heatmap shown in (A). (F–I) Orthogonal validation for compound B using Western blot analyses of *p*-EGFR and *p*-ERK1/2 (measured in A549 cells) (F) and *p*-ERBB4 and *p*-ERK1/2 (measured in T-47 cells) (G) using increasing concentrations of compound B (Cpd B). (H) Quantification of relative *p*-EGFR and *p*-ERBB4 from (F, G). (I) Quantification of relative *p*-ERK1/2 from (F, G). (J and K) *In vitro* kinase activity assays using LANCE assays for compound A (J) and compound B (K) showing dose response graphs comparing drug selectivity for ERBB4 (red) and EGFR (black). (L) Dose response graphs for CRE sensor responses in PC12 cells using luciferase as readout for compounds A (black) and B (red). In addition to the increasing concentrations of the compounds, the constant stimulation mix as in (A) was present. (M and N) Dose response graphs for calcium and cAMP assays using Fluo-4 a.m. (black) and GloSensor (red), respectively, as readouts in PC12 cells treated with increasing concentrations of compound A (M) and compound B (N). In addition to the compounds, the constant stimulation mix was present as in (A). Error bars represent SEM; n = 3 for barcode assays (B–E), Western blots (F–I) and *in vitro* kinase assays (J, K); n = 6 for luciferase assays, Fluo-4 a.m., and GloSensor assays (L–N). See also Figures S4 and S5.

Next, we sought to profile potentially selective ERBB4 antagonists, termed compounds A and B, that were designed and synthesized in a proprietary kinase inhibitor program to challenge our barcoded ERBB family profiling assay. The main goal was to identify compounds that show a selectivity for ERBB4 over EGFR for the potential use in follow-up drug discovery campaigns. Both compounds A and B more efficiently inhibited ERBB4 than EGFR (Figure 5A). When extracting the data from this heatmap and computing dose-response curves, we found that both compounds showed a higher activity for ERBB4 than for EGFR and to some extent for ERBB2/3 (Table S3). Importantly, compound B had both better efficacy and preference for ERBB4 over EGFR (ERBB4 IC_50_: 0.142 μM, EGFR IC_50_: 1.085 μM, about 8-fold) than compound A (ERBB4 IC_50_: 0.575 μM, EGFR IC_50_: 1.146 μM, about 2-fold) (Figures 5B and 5C), defining compound B as frontrunner compound. Similarly, ERK signaling was inhibited by these two compounds in an EGFR, ERBB2/3, and ERBB4 mediated manner, reflecting the inhibition at receptor level (Figures 5D and 5E). Biochemical validation by Western blotting of phospho-EGFR (in A549 cells) (Figure 5F) and phospho-ERBB4 (in T-47D cells) (Figure 5G) confirmed that compound B preferentially antagonized ERBB4, as revealed by quantification of phosphorylation levels (Figure 5H). An analysis of downstream phospho-ERK1/2 showed that compound B reduced the ERK pathway activity to about 70% of activated EGFR and ERBB4 (Figure 5I). To confirm that compounds A and B inhibited EGFR and ERBB4 at different potencies, we conducted LANCE kinase activity assays. In this *in vitro* executed enzymatic assay, synthetic substrates are incubated with EGFR and ERBB4 purified protein, and the amount of phosphorylated substrate is detected by a specific anti-phosphopeptide antibody, resulting in a time resolved fluorescence resonance energy transfer (TR-FRET) signal. Notably, both compounds A and B inhibited ERBB4 more effectively than EGFR (Figures 5J and 5K). In addition, compound B displayed a better preference profile for ERBB4 than EGFR, as evident from IC_50_ values for compound B (ERBB4: 0.059, EGFR: 0.525) and compound A (ERBB4: 0.01, EGFR: 0.067). Thus, compound B showed an about 9-fold preference for ERBB4, while compound A only displayed an about 7-fold preference, validating the findings of our cellular profiling assay that compound B had better selectivity for ERBB4.

An analysis of downstream pathway activities further showed that compound A treatment at 10 μM led to the activation of the CRE sensor for all targets tested (Figure 5A). A separate validation assay in PC12 cells by transfecting only a CRE sensor linked to luciferase readout confirmed this opposite behavior of these two novel ERBB4 antagonists. In this assay, PC12 cells were treated with increasing concentrations of either compound A or B and the stimulation mix containing EGF, EGFld, and serotonin to mimic the conditions from the barcoded assays. While compound A treatment led to increased CRE activity at concentrations of ≥3 μM, compound B treatment inhibited CRE activity in a dose-dependent manner (Figure 5L). To assess whether the CRE sensor is modulated by altered levels of cAMP or calcium in response to ERBB4 antagonist treatment, the GloSensor assay was used to measure cAMP and Fluo-4 a.m. to assess calcium. As in luciferase validation assays, PC12 cells were treated with increasing concentrations of either compound A or B and the stimulation mix. Calcium levels were neither affected by compound A nor by compound B. However, compound A concentrations of ≥3 μM caused an increase in cAMP levels, while compound B treatment, starting with 1 μM and higher, caused a steady decrease in cAMP, mirroring the effects from the CRE sensor barcode and luciferase assays (Figures 5M and 5N). Furthermore, treatment with compound B, but not compound A, resulted in an inhibition of HTR2A at target (split TEV assay) and pathway levels (CRE assay) in PC12 cells (Figures 5A, S5A, and S5B), a finding that was validated by independent luciferase assays for compound B (Figure S5C). Taken together, we identified two novel ERBB4 selective antagonists, with compound B being more selective and, at least in cell culture, more potent than compound A.

## Discussion

We describe the establishment of a barcoded ERBB receptor profiling assay that enables the simultaneous profiling of compound actions on ERBB targets and key downstream pathways in living cells. Specifically, split TEV assays for monitoring the activities of full-length EGFR, ERBB2/3, ERBB4, and HTR2A (a GPCR as non-ERBB receptor control) are parallelized with pathway assays for cAMP/Ca^2+^ signaling (CRE sensor), pure Ca^2+^ signaling (NFAT sensor), and the ERK branch of MAPK signaling (EGR1p sensor) in this multiparametric assay platform. Notably, downstream pathway responses were uniquely assigned to each receptor, allowing the identification of functionally distinct activities when treated with a compound from one well. For example, while the MAPK sensor EGR1p was inhibited by pyrotinib, the CRE sensor was activated at high concentrations for all ERBB receptors. Furthermore, varying intensities of inhibition or activation for one pathway were clearly assigned to a specific ERBB receptor activity. Barcoded assays are as responsive and robust as single luciferase assays, as evidenced by similar EC_50_ values obtained from these two assay types for targets and pathways tested. Through applying a multilevel barcoding strategy for analyzing cellular activities (i.e., sensors with unique barcodes for targets and pathways), wells (sample barcoding through pooling of cell lysates using Tag&Pool and PCR-based barcoding), and cell culture plates (PCR-based barcoding) in parallel, we reduced sample numbers and hands-on time for sample processing and NGS analyses, while processing thousands of data points. In addition, we reduced sample numbers in drug profiling assays even further by stimulating ERBB receptor and HTR2A targets with a ligand mix. The principle of the barcoded profiling assay can be extended in the future to any other RTK, GPCR, or other targets for which split TEV assays are available or can be developed. Pathway sensors used here can be applied to any RTK or GPCR assay, and additional sensors can be developed to capture activities of other cellular pathways. However, the number of pathway sensors possibly used per target is limited by the well size (i.e., number of cells that fit into a well), the number of targets used per well, and the depth of the sequencing, thus affecting the overall assay’s complexity.

Commonly used kinase profiling assays for ERBB receptors are based on biochemical kinase platforms, which can be divided into two classes, activity assays and binding assays. Activity assays directly or indirectly measure the catalytic product and include radiometric platforms (e.g., HotSpot),^63^ luminescence-based (e.g., Kinase-Glo), and fluorescence-based platforms (e.g., Lance TR-FRET assay or LanthaScreen TR-FRET assay).^64^ (Note that compounds A and B were validated using the Lance TR-FRET kinase activity assay.) In contrast, binding assays measure the binding of compounds to the kinase active site, but not the catalytic product (e.g., KinomeScan).^64^ All of these are single assays and biochemical in nature. A more focused platform for the analysis of RTK activities uses a membrane-based sandwich immunoassay with phosphotyrosine antibodies and some degree of multiplexing as one cellular lysate can be used to profile 49 different RTKs.^65^ RTKs and cellular downstream effectors can also be profiled using an image-based approach, as described for EGFR.^66^ This method was reported to be robust but rather labor intensive, making multiplexing inefficient. Although most of these assays use smaller well formats than we did in this study, multiplexing at the assay level (including both targets and pathways), well level, and plate level as introduced for our multilevel barcoded reporter profiling assay is not feasible. In contrast, using NGS as a readout for our barcoded assay, we simultaneously monitored the effects of compounds on ERBB receptors and their downstream pathways, using 50 assays per well and obtaining 12,000 data points from a standard assay setup.

We challenged the ERBB receptor profiling assay with eight different ERBB receptor antagonists (AG1478, erlotinib, gefitinib, lapatinib, osimertinib, poziotinib, pyrotinib, and TAS6417) that were developed to target different types of cancers. All these drugs inhibited the activity of EGFR, ERBB2/3, ERBB4 at different levels, and likewise, canonical downstream ERK signaling. When systematically comparing our IC_50_ data with published data obtained from cellular assays, we noticed that we had comparable potencies for some of the eight ERBB antagonists on selected targets and pathways,^28^^,^^48^^,^^51^^,^^52^^,^^53^ while for other combinations our potency values were an order of magnitude higher (cf. Table S4).^49^^,^^52^^,^^54^^,^^55^^,^^56^ The latter case may be due to different cell types and/or assay systems applied. Nevertheless, a substantial number of studies used phospho-blots to demonstrate the drug effect on ERBB receptors. Although this is a very reliable technique, due to its technical demands, most of these findings were only qualitative in nature, especially when studying pathway activities. Conversely, our ERBB profiling assay allows the monitoring of multiple compounds on ERBB receptors and linked pathways in parallel, saving both hands-on time and money. We validated our previous findings for lapatinib that showed a preference for ERBB2/3 and ERBB4 over EGFR,^37^ while spironolactone was an ERBB4 selective antagonist.^32^ Most strikingly, AG1478 displayed a preference for EGFR, while pyrotinib showed a preference for ERBB4. As this was expected for AG1478,^67^ pyrotinib’s effect on ERBB4 is novel. Together, these features verify that the barcoded ERBB receptor profiling assay can detect both preferred activities of compounds for EGFR versus ERBB4 and represents an optimal route to assess ERBB antagonists in living cells. The ERBBprofiler assay enables the simultaneous monitoring of target and pathway effects in living cells. In this respect, we identified pathway characteristics for AG1478, as we quantified its effect on ERBB4 and MAPK signaling downstream of EGFR for the first time *in cellulo*. Likewise, we monitored effects of TAS6417 on ERBB2/3 and ERBB4 for the first time *in cellulo*.^57^^,^^68^^,^^69^ Poziotinib was the only covalent inhibitor we tested, and it is known to inhibit ERBB receptor activities at nanomolar range.^53^^,^^70^ Indeed, we observed a very strong activity of this drug, both at target and pathway level, arguing that our platform reliably detects compound effects across several magnitudes of molar range.

Frequently, we observed enhanced potency of drugs on pathway assays (i.e., lower IC_50_ values) when compared to target assays, suggesting that compound effects are amplified within the process of cellular signaling.^71^ Conversely, target assays were more useful in identifying a drug’s selectivity for a given ERBB receptor. Importantly, activity trends for targets aligned well with pathway activities in our profiling assay, supporting the notion that both assay types can be used to assess compound actions. Nevertheless, for some conditions, drug effects on targets may be very subtle or even hidden,^72^^,^^73^ and pathway assays may be used to unveil these effects through cellular signal amplification.

At high drug concentrations of 10 μM, we observed an upregulated CRE sensor response for many of the tested drugs (i.e., erlotinib, gefitinib, lapatinib, osimertinib, poziotinib, pyrotinib, TAS6417, canrenone, and compound A). Using orthogonal assays specific for cAMP and calcium we found that high concentrations of poziotinib treatment led to an increase of cAMP. Increased cAMP concentrations are linked to cellular stress pathways, cellular energy status, as well as oxidative and proteotoxic stress,^14^^,^^74^^,^^75^^,^^76^ suggesting that high concentrations of selected ERBB antagonist are the cause for this abnormal cellular signature. Remarkably, clozapine, spironolactone, and compound B did not activate the CRE sensor at 10 μM, suggesting that these compounds are also better tolerated at higher, albeit non-physiological, concentrations. In fact, PC12 cells treated with compound B, and without transfecting any other plasmids than the GloSensor, resulted in a concentration-dependent decrease of cAMP, which might be mediated by antagonizing activities conferred by HTR6A, a Gα_s_ protein coupled GPCR expressed in PC12 cells,^77^ or by inhibiting components of the protein kinase A/cAMP pathway.

Increased ERBB4 activity leads to an excitation/inhibition imbalance in schizophrenic patients,^30^^,^^31^ and antagonizing ERBB4 activity using spironolactone improved schizophrenia relevant phenotypes including cognition in a *Nrg1* transgenic mouse model,^32^ prompting also a clinical trial with spironolactone as adjuvant to antipsychotic medication, i.e., co-administering spironolactone with an established antipsychotic, e.g., risperidone or aripiprazole.^78^ Notably, a separately conducted clinical trial using spironolactone as add-on therapeutic to risperidone reported improvements for positive and negative symptoms, but not cognitive deficits.^79^ In addition, spironolactone as mineralocorticoid receptor antagonist was shown to improve cognition in mouse models of type II diabetes^80^ and Alzheimer’s disease.^81^ In our profiling assay, we found that spironolactone also inhibits HTR2A, and antagonists to HTR2A are frequently used for the treatment of schizophrenia,^82^ suggesting that such a polypharmacological profile may be beneficial for alleviating symptoms in schizophrenic patients.

When targeting ERBB4, at least in the CNS, a drug’s selectivity over EGFR is most crucial, as EGFR activity is key for the propagation of neural precursors and the differentiation of these into neurons, while ERBB4 is linked to schizophrenic phenotypes.^83^^,^^84^ In addition, ERBB4 is most closely related to EGFR.^85^^,^^86^ A recent study reported two investigational compounds that are ERBB4-selective over EGFR, with a 2.5-fold pref. 87. However, compounds with a higher selectivity are required to bypass any non-desired effects that may be mediated through EGFR inhibition. Here, we identify two novel compounds that were synthesized in a proprietary kinase inhibitor program and that have an increased selectivity for ERBB4 over EGFR, with compound B displaying an about 8-fold preference. Importantly, this strong preference for ERBB4 for compound B we observed in the cellular profiling assay was confirmed by *in-vitro* kinase assays, albeit to a lesser degree. Furthermore, compound B, but not compound A also inhibited HTR2A. Therefore, compound B with its pharmacological profile may represent a lead structure for a therapeutic application targeting schizophrenia in stratified patients with an ERBB4 dependent cognitive deficit, requiring further work on pharmacokinetics, pharmacodynamics, elucidating the mechanism of action, and the compound’s effect on behavior in mice. Given a sufficient compound availability in the brain, this novel ERBB4 antagonist should be tested in an add-on study paradigm to assess any improvement in behavioral tests in a relevant mouse model.^88^^,^^89^ Moreover, ERBB4 overexpression is associated with numerous cancer types (e.g., gastric cancer, non-small cell lung cancer, inflammatory breast cancer, pancreatic cancer, prostate cancer).^17^^,^^90^^,^^91^^,^^92^ Thus, the newly developed compounds A and B may also be lead structures for targeting ERBB4 positive cancers.

In summary, we have developed a multiplexed cell-based profiling assay, termed ERBBprofiler assay that assesses drug effects on ERBB family receptors and key cellular signaling pathways, opening an efficient route for early-stage drug discovery of ERBB antagonists. Using this assay, we described previously unknown compound properties for selected ERBB receptor inhibitors. Furthermore, we identified newly designed ERBB4 selective antagonists that have an increased selectivity and could be used as lead compounds in drug discovery programs. Due to the multilevel barcoding approach, multiple assays and treatment parameters can be run in parallel, enabling the acquisition of thousands of data points from one experiment. Stable integration of assay components into cells, expansion to additional targets, and automation through liquid handling robotics is expected to further enhance the assay robustness and throughput to enable profiling of larger compound collections.

### Limitations of the study

For the ERBBprofiler assay, we focused on monitoring the activities of EGFR, ERBB2/3, and ERBB4. We did not measure the effects of ERBB2 and ERBB3 homodimers, although they could be added to an extended version of the ERBBprofiler assay. We included pathway sensors for three key downstream pathways of ERBB receptors, namely cAMP signaling, Ca^2+^ signaling, and the ERK branch of MAPK signaling. However, we excluded other ERBB receptor-regulated pathways, such as AKT signaling. In the future, sensors for AKT signaling and other relevant signaling pathways may also be added. The complexity of the ERBBprofiler assay is determined by the number of barcoded assays in each well. Here, the ERBBprofiler assay consisted of 50 individual assays per well and was performed in 24-well formats. We did not test whether the ERBBprofiler assay with a complexity of 50 assays also performs robustly in smaller well formats, like 48-well and 96-well formats, to enable a high-throughput compatible screening of compounds. Therefore, the current form of the ERBBprofiler assay is constrained to a medium throughput format that uses 24-well plates. Furthermore, it is feasible to introduce additional assays for both the receptors and pathways that would increase the complexity per well. However, this could have an adverse effect on the performance of the assay when conducted in a 24-well format, as utilized in this study. If more sensors will be added per well, more cells per well may be required for the efficient sequencing of barcoded reporters. We conducted all experiments using transient transfections. Assays with a transient transfection may, however, have a reduced robustness and reproducibility compared to stably integrated assay components. Therefore, our future investigations will primarily focus on making cell lines with receptors that have been stably integrated.

## STAR★Methods

### Key resources table

**Table:** 

REAGENT or RESOURCE	SOURCE	IDENTIFIER
**Antibodies**		

Rat monoclonal anti-HA High Affinity (clone 3F10)	Roche	Cat# 11867423001; RRID: AB_390918
Rabbit monoclonal anti-phospho-EGF Receptor (Tyr1068) (clone D7A5)	Cell Signaling Technology	Cat# 3777; RRID: AB_2096270
Rabbit monoclonal anti-phospho-HER4/ErbB4 (Tyr1284) (clone 21A9)	Cell Signaling Technology	Cat# 4757: RRID: AB_2099987
Mouse monoclonal anti-EGFR (clone A-10)	Santa Cruz Biotechnology	Cat# sc-373746: RRID: AB_10920395
Rabbit monoclonal anti-HER4/ErbB4 (clone E200)	Abcam	Cat# ab32375; RRID: AB_731579
Rabbit monoclonal anti-phospho-p44/42 MAPK (Erk1/2) (Thr202/Tyr204) (clone D13.14.4E)	Cell Signaling Technology	Cat# 4370; RRID: AB_2315112
Rabbit monoclonal anti-p44/42 MAPK (Erk1/2) (clone 137F5)	Cell Signaling Technology	Cat# 4695; RRID: AB_390779
Mouse monoclonal anti-α-tubulin (clone B-5-1-2)	Sigma-Aldrich	Cat# T5168; RRID: AB_477579
Eu-Anti-Phosphotyrosine (PT66) Antibody (AB)	PerkinElmer	Cat# AD0068

**Bacterial and virus strains**		

One Shot Mach1 T1 Phage-Resistant Chemically Competent *E. coli*	Thermo Fisher Scientific	Cat# C862003

**Chemicals, peptides, and recombinant proteins**		

AG-1478 (Tyrphostin AG-1478)	Selleckchem	Cat# 2728; CAS: 153436-53-4
Canrenone	Santa Cruz Biotechnology	Cat# sc-205616; CAS: 976-71-6
Clozapine	Sigma-Aldrich	Cat# C6305; CAS: 5786-21-0
EGFR	Thermo Fisher Scientific	Cat# PV4190
ErbB4	Thermo Fisher Scientific	Cat# PV4104
Erlotinib hydrochloride	Sigma-Aldrich	Cat# SML2156; CAS: 183319-69-9
Gefitinib	Sigma-Aldrich	Cat# SML1657; CAS: 184475-35-2
Lapatinib (GW-572016) Ditosylate	Selleckchem	Cat# S1028; CAS: 388082-77-7
Osimertinib (AZD9291)	Selleckchem	Cat# S7297; CAS:1421373-65-0
Poziotinib (HM781-36B)	Selleckchem	Cat# S7358; CAS: 1092364-38-9
Pyrotinib	MedChemExpress	Cat# HY-104065; CAS: 1269662-73-8
Spironolactone	Selleckchem	Cat# S4054; CAS: 52-01-7
TAS6417 (Zipalertinib)	Selleckchem	Cat# S8814; CAS: 1661854-97-2
hEGF	Sigma-Aldrich	Cat# E9644; CAS: 62253-63-8
Heregulin-β1 (EGF Domain) human (EGF-like domain)	Sigma-Aldrich	Cat# H7660
Serotonin hydrochloride	Tocris Bioscience	Cat# 3547; CAS: 153-98-0
Compound A	This paper; Lead Discovery Center	N/A
Compound B	This paper; Lead Discovery Center	N/A
ATP	Sigma	Cat# A7699
LR Clonase™ II Plus enzyme	Thermo Fisher Scientific	Cat# 11538120

**Critical commercial assays**		

GloSensor Technology	Promega	Cat# E2301
Fluo-4, AM, cell permeant	Thermo Fisher Scientific	Cat# F14201
LANCE Ultra ULight™-poly GT	PerkinElmer	Cat# TRF0100
LANCE Ultra ULight™-JAK-1 (Tyr1023) Peptide	PerkinElmer	Cat# TRF0121
NovaSeq 6000 SP Reagent Kit v1.5 (100 cycles)	Illumina	Cat# 20028401
NovaSeq 6000 SP Reagent Kit v1.5 (200 cycles)	Illumina	Cat# 20040719

**Deposited data**		

Analyzed data	This paper	Mendeley Data: https://doi.org/10.17632/vxyy62m2c7.1
Data of barcoded ERBBprofiler assays	This paper	Mendeley Data: https://doi.org/10.17632/vxyy62m2c7.1
Data of orthogonal validation assays (standard split TEV and reporter gene assays, Fluo-4 AM calcium assays, cAMP GloSensor assays, quantification of WB assays)	This paper	Mendeley Data: https://doi.org/10.17632/vxyy62m2c7.1
DRC R script	This paper	Mendeley Data: https://doi.org/10.17632/vxyy62m2c7.1
Heatmap R script	This paper	Mendeley Data: https://doi.org/10.17632/vxyy62m2c7.1

**Experimental models: Cell lines**		

*Rattus norvegicus*: PC12 Tet-Off cells	Clontech	Cat# 631134; RRID: CVCL_V361
Human: A-549 cells	ATCC	Cat# CCL-185; RRID: CVCL_0023
Human: T-47D cells	ATCC	Cat# HTB133; RRID: CVCL_0553

**Oligonucleotides**		

See Table S7	This paper	N/A

**Recombinant DNA**		

See Table S4	This paper	N/A

**Software and algorithms**		

ImageJ	Schneider et al., 2012^93^	https://imagej.nih.gov/ij/
R version 4.2.3 or higher	R Core Team (2023)	https://www.R-project.org/
RStudio2023.03.0 + 386	RStudio Team (2023)	http://www.rstudio.com/
ggplot2	Wickham, 2016^94^	https://ggplot2.tidyverse.org
reshape	Wickham, 2007^95^	http://www.jstatsoft.org/v21/i12/paper
tidyr	Wickham et al., 2023^96^	https://CRAN.R-project.org/package=tidyr
tidyverse	Wickham et al., 2019^97^	https://doi.org/10.21105/joss.01686
drc	Ritz et al., 2015^98^	http://journals.plos.org/plosone/article?id=10.1371/journal.pone.0146021
BioRender	BioRender.com (2023)	https://app.biorender.com/biorender-templates
Adobe Illustrator CS6	Adobe Inc.	https://adobe.com/products/illustrator
MikroWin 2000 Version 4.41	Mikrotek Laborsysteme (1992–2007)	https://mikrowin-2000.software.informer.com/download/
OPTIMA Version 2.20R2	BMG Labtech	https://www.bmglabtech.com/en/microplate-reader-software/
ChemoStar Imager	Intas Pharmaceuticals	https://www.intas.de/chemilumineszenz-westernblotting/73-chemocam-imager
Echo Dose Response Software	Beckman Coulter	https://www.beckman.de/liquid-handlers/software/echo/dose-response

### Resource availability

#### Lead contact

Further information and requests for resources and reagents should be directed to and will be fulfilled by the Lead Contact, Michael Wehr (michael.wehr@med.uni-muenchen.de).

#### Materials availability

Plasmids used for standard split TEV assays and standard reporter gene assays are available from Systasy Bioscience GmbH (www.systasy.de). There are restrictions to the availability of the reporter plasmids used for the ERBBprofiler assay due to a material transfer agreement (MTA). There are restrictions to the availability of the two compounds A and B due to intellectual property considerations.

#### Data and code availability

• Data of barcoded ERBBprofiler assays and orthogonal validation assays have been deposited at Mendeley Data and is publicly available as of the date of publication. DOIs are listed in the key resources table.
• Original code has been deposited at Mendeley Data and is publicly available as of the date of publication. DOIs are listed in the key resources table.
• Any additional information required to reanalyze the data reported in this paper is available from the lead contact upon request.

### Experimental model and study participant details

#### Cell lines

PC12 Tet-Off cells (RRID:CVCL_V361) (Clontech, 631134, termed PC12 cells for simplicity) were maintained in DMEM medium (1 g/L glucose, Lonza) supplemented with 10% FBS, 5% horse serum (HS, Thermo Fisher Scientific Inc.), and 100 U/ml each of penicillin and streptomycin (Thermo Fisher Scientific Inc., Cat. No. 15140-122) and 2 mM GlutaMAX (Thermo Fisher Scientific Inc., Cat. No. 35050038). Starvation of PC12 cells was induced by 1% FCS, 100 U/ml each of penicillin and streptomycin and 2 mM GlutaMAX, but no HS. Cell-based assays for PC12 cells were performed in starvation conditions. A549 cells (RRID:CVCL_0023) (ATCC, CCL-185) were cultured in DMEM medium (4.5 g/L glucose) supplemented with 10% FCS and 100 U/ml each of penicillin and streptomycin and 2 mM GlutaMAX. T-47D cells (RRID:CVCL_0553) (ATCC, HTB-133) were cultured in RPMI 1640 medium supplemented with human insulin (f.c. 125 μg/L) (Sigma-Aldrich), 10% FCS and 100 U/ml each of penicillin and streptomycin and 2 mM GlutaMAX.

#### Plasmids

Plasmids for EGFR, ERBB3, and ERBB4 fused to NTEV-tcs-GV moieties and ERBB2 fused to a V5-tag were previously described,^32^ as well as for HTR2A and ARBB2,^35^ and the clustered SH2 domains of GRB2.^37^ For the cloning of pathway reporters for CRE, EGR1p, and NFAT elements, the Gateway Destination vector pGL4.16_attR1_Insert_attR2_luc2/Hygro_DEST (described in^35^ and based on the pGL4.16_luc2/Hygro vector from Promega) was used. Entry vectors for CRE (pENTR/221_attL1-CRE-attL4, contains 6 CRE repeats), EGR1p (pENTR/221_attL4r_EGR1p_attL3r, contains a 1.0 kb promotor region of the human EGR1 promoter), NFAT (pENTR/221_attL1_NFAT-RE_attL4, contains 6 NFAT repeats), a dummy Entry vector for attL1-attL4 sites (pENTR/221_attL1_dummy_attL4) and an Entry vector carrying a unique 49-mer barcode sequence (pENTR/221_attL3_BarcodeLibrary_attL2) were obtained from a previous study.^99^ pcDNA3.1(+) was obtained from Thermo Fisher Scientific. All plasmids used in this study are listed in Table S1.

#### Bacterial strains

The Mach1 competent cells were purchased from Thermo Fisher Scientific and used as competent cells to construct the vectors.

#### Chemical reagents

The following commercial compounds were used in this study: AG1478 (Selleckchem, S2728), canrenone (Santa Cruz, sc-205616), clozapine (Sigma-Aldrich, C6305), erlotinib (Sigma-Aldrich, SML2156), gefitinib (Sigma-Aldrich, SML1657), lapatinib (Selleckchem, S1028), osimertinib (Selleckchem, S7297), poziotinib (Selleckchem, S7358), pyrotinib (MedChemExpress, HY-104065), spironolactone (Selleckchem, S4054), TAS6417 (Selleckchem, S8814), EGF (Sigma-Aldrich, E9644, EGF-like domain (Sigma-Aldrich, H7660), serotonin (Tocris, 3547). Compounds A and B were synthesized by the Department of Medicinal Chemistry of the Lead Discovery Center, Dortmund, Germany, as part of a proprietary kinase inhibitor program.

### Method details

#### Vector construction

Pathway reporters for CRE, EGR1p, and NFAT elements were cloned using a Multisite Gateway recombination (Thermo Fisher Scientific) strategy as described in^99^ and linked to a firefly luciferase gene and unique barcodes for multiparametric assays. Entry vectors harboring CRE and NFAT sensors were combined with an Entry vector harboring an adenovirus major late promoter (MLP) (pENTR/221_attL4r_MLP_attL3r) and an Entry vector carrying a unique 49-mer barcode (pENTR/221_attL3_Barcode_attL2). The EGR1p Entry vector (pENTR/221_attL4r_EGR1p_attL3r) was combined with the dummy Entry vector (pENTR/221_attL1_dummy_attL4) and a unique 49-mer barcode Entry vector (pENTR/221_attL3_BarcodeLibrary_attL2). Each of the sets containing three Entry vectors were recombined using LR Clonase II Plus enzyme (Thermo Fisher Scientific) into pGL4.16_attR1_Insert_attR2_luc2/Hygro_DEST to yield expression vectors, which are listed in Table S1. The sequences of the expression plasmids were verified using Sanger sequencing.

#### Luciferase reporter assays

50,000 PC12 cells were seeded per 96-well onto poly-L-lysin (PLL)-coated plates and transfected the following day (day *in vitro* 1, DIV1) with assay plasmids using Lipofectamine 3000 (Thermo Fisher Scientific). For split TEV assays, plasmids (pcDNA3 or pTag4C backbone) encoding receptor-NTEV-tcs-GV, GRB2-SH2-CTEV (for ERBB receptors) or ARBB2-1-383-CTEV (for HTR2A) (all 10 ng/well), and the p10xUAS-luc2 reporter plasmid (10 ng/well) were used. For pathway assays, the receptor plasmids (15 ng/well) and pathway reporter plasmids (15 ng/well) pEGR1p-luc2 for monitoring MAPK signaling, pCRE-CMVmin-luc2 for monitoring cAMP/Ca^2+^ signaling, or p6xNFAT-CMVmin-luc2 for Ca^2+^ signaling were used. Combinations of assays conducted are summarized in Table S5. Transient transfections were conducted according to the manufacturer’s instructions. On DIV2, cells were starved in medium containing 1% FCS for 16 h. On DIV3, a stimulus was added at increasing doses or, for antagonist assays, at single concentrations (30 ng/mL EGF for activating EGFR; 10 ng/mL EGFld for activating ERBB2/3 and ERBB4; and 1 μM serotonin for activating HTR2A) for 6 h. In the case of antagonist assays, compounds were added at increasing doses together with the stimulus. Cells were lysed in 1x passive lysis buffer (Promega) and subjected to a firefly luciferase assay using a self-made substrate for firefly luciferase.^100^ Luciferase activity was analyzed in a Mithras LB 940 Multimode Microplate Reader (Berthold Technologies). All assays were run in 96-well plates using six replicates per condition.

#### GloSensor cAMP assay

The GloSensor cAMP assay was conducted with the same parameters (50,000 PC12 cells per 96-well) as described above for the firefly luciferase assay, with however, three differences. Specifically, (1) 30 ng/well of the pGloSensor-22F plasmid (Promega, E2301) was used for transfection, and (2) on DIV3, cells were equilibrated in assay medium containing a 2% v/v dilution of D-luciferin (Synchem, bc219) for 2 h at 37°C before (3) the treatment of compounds for 15 min and lysis in 1x passive lysis buffer (Promega) and luciferase activity measurement.

#### Fluo-4 a.m. calcium assays

50,000 PC12 cells were seeded per 96-well onto poly-L-lysin (PLL)-coated clear bottom plates (PerkinElmer, 6055302) and starved on DIV1 for 24 h. On DIV2, cells were pre-equilibrated in 10 μM Fluo-4 a.m. (Thermo Fisher Scientific, F14201) away from light for 1 h at 37°C. Next, cells were incubated in the indicator-free starvation medium to allow de-esterification for 20 min away from light at 37°C. Background fluorescence was then measured using the BMG POLARstar Optima Microplate Reader prior to adding the treatments and continuously measuring the fluorescence for the next 30 min. Fluorescence was measured using the bottom optics.

#### Barcode reporter assays

##### Transfection of reporter plasmids and stimulation conditions

Mulitplexed barcoded reporter assays were conducted in PC12 cells using 24-well plates, with 3 replicates per condition. Per well, 250,000 PC12 cells were transfected *in solution* with assay plasmids using Lipofectamine 3000. Per well, 5 batches of transfected cells were used. Per well, amounts of assay plasmids were 33 ng of target and adapter plasmids, and 27 ng of each reporter plasmid (note that 10 reporters were used per batch), totaling to 336 ng/well. Combinations of barcoded assays conducted are summarized in Table S6, barcode sequences used as RNA reporters are listed in Table S1. Transfected cells were incubated for 2 h at 37°C and 5% CO_2_. Tubes (15 mL or 50 mL Falcon tubes) containing transfected cells were loosely closed to allow the CO_2_ flow and put tilted at 45° angle to enhance transfection efficiency and viability. Cells were then centrifuged for 5 min at 1000 rpm, the whole supernatant was carefully aspirated, and cells were plated in maintenance medium into PLL-coated 24-well plates for 24 h. Cells were then starved for 16 h as described above and treated for 6 h with compounds. Agonists and antagonists were applied simultaneously. Cells were lysed in 400 μL/well in a wash and lysis buffer (100 mM Tris/HCl pH 7.5, 500 mM LiCl, 10 mM EDTA, 5 mM DTT, 1% LiDS). Plates were shaken for 10 min at 200 rpm for thorough lysis.

##### Isolation of barcodes, library preparation, and next-generation-sequencing

For Tag&Pool, a procedure to combine multiple cell lysates for single purification and processing of barcode reporter RNAs, second level barcodes (to track wells, see Figures 1 and S1I) were added to the lysates to a final concentration of 0.125 μM for annealing at 65°C for 15 min. Once cooled to room temperature, all 24 lysates from one 24-well plate and 20 μL of M-PVA OdT2 beads (Chemagen, Cat. No. CMG-231) were pooled. Next, beads were subjected to a series of wash steps, including one wash with 100 μL of 1x High-Capacity reaction buffer (High-Capacity cDNA Reverse Transcription Kit, Thermo Fisher Scientific, Cat. No. 4368814). Finally, cDNA synthesis from beads was performed in 20 μL of cDNA High-Capacity cDNA Reverse Transcription mix at 25°C for 25 min. For barcoded (‘tagged’) cDNA amplification, a forward primer containing the Read1 Illumina adapter sequence and an UMI sequence was used, in combination with a Read2 reverse primer (30 PCR cycles). Illumina indices and sequencing adapters were attached (10 PCR cycles), the final barcode libraries were pooled in equimolar ratio (2 p.m.) and subjected to paired end, dual index sequencing with the NovaSeq 6000 SP Reagent Kit v1.5 (Illumina GmbH, Cat. No. 20028401 or 20040719). Oligos used are listed in Table S7.

#### Biochemistry

Phosphorylation levels of EGFR were assessed^37^ in A549 cells, while phosphorylation levels of ERBB4 were assessed in T-47D cells. Before stimulation experiments were performed, both cell types were starved overnight in 1% FCS and compounds were incubated for 1 h. A549 were stimulated with 30 ng/mL EGF for 5 min, while T-47D cells were stimulated with 10 ng/mL EGFld for 5 min. For lysis, cells were washed 1x with PBS and lysed in a Triton X- lysis buffer (1% Triton X-100, 50 mM Tris pH7.5, 150 mM NaCl, 1 mM EGTA) containing the Complete protease inhibitor cocktail (Roche) and PhosSTOP phosphatase inhibitor (Roche). Briefly, cells were lysed and kept on ice for 10 min, sonicated 3x for 10 s at 4°C, and denatured for 10 min at 70°C. The Mini-PROTEAN Tetra Electrophoresis System and Trans-Blot Turbo Botting System (both Bio-Rad) were used for running and blotting protein gels. Chemiluminescence detection of proteins by Western blot analysis was performed using the Western LightningPlus-ECL kit (PerkinElmer). HA-tagged proteins were visualised using an HA antibody (RRID: AB_390918) (clone 3F10, dilution 1:1000, No. 11 867 423 001, Roche). Phosphorylation levels of EGFR and ERBB4 were assayed using *p*-EGFR-Y1068 (RRID: AB_2096270) (clone D7A5, dilution 1:500, No. 3777, Cell Signaling Technology) and *p*-ERBB4-Y1284 antibodies (RRID: AB_2099987) (clone 21A9, dilution 1:500, No. 4757, Cell Signaling Technology). Total EGFR and ERBB4 protein levels were determined using an anti-EGFR antibody (RRID: AB_10920395) (clone A-10, dilution 1:1000, sc-373746, Santa Cruz Biotechnology) and an anti-ERBB4 antibody (RRID: AB_731579) (clone E200, dilution 1:1000, ab32375, Abcam). Phosphorylation levels of ERK1/2 were assayed using p-p44/42 MAPK (Erk1/2) (RRID: AB_2315112) (clone D13.14.4E, dilution 1:5000, No. 4370, Cell Signaling Technology), total ERK1/2 protein levels were assayed using p44/42 MAPK (Erk1/2) (RRID: AB_390779) (clone 137F5, dilution 1:5000, No. 4695, Cell Signaling Technology). Tubulin levels were determined using an anti-Tubulin antibody (RRID: AB_477579) (dilution 1:2000, No. T 5168, Sigma-Aldrich). For quantification, phosphorylation levels of *p*-EGFR relative to EGFR as well as *p*-ERBB4 relative to ERBB4 were calculated using the Lukemiller protocol (http://lukemiller.org/index.php/2010/11/analyzing-gels-and-western-blots-with-image-j/). Assays were run in triplicates.

#### *In vitro* kinase assays

*In vitro* kinase assays for compounds A and B were conducted using the LANCE Ultra TR-FRET kinase activity assays for EGFR and ERBB4. The principle of this enzymatic assay is based on the phosphorylation of the U*light*-peptide substrate, labeled with an acceptor fluorophore, by a purified protein kinase. Phosphorylation is detected by a specific Europium-labelled anti-phospho-peptide antibody, labeled with a donor fluorophore. The binding of the Europium-labelled anti-phospho-peptide antibody to the phosphorylated U*light*-labelled peptide produces an FRET signal. Binding of an inhibitor to the kinase prevents phosphorylation of the U*light*-substrate, resulting in a loss of FRET. For every sample, 2 μL of assay buffer (50 mM HEPES pH 7,5, 10 mM MgCl_2_, 1 mM EGTA 0.01% Tween 20, 1% DMSO, 2 mM DTT) were transferred into a 384-well plate (Corning #4513). Compounds were added in a concentration range from 10 μM to 0.0025 μM using an acoustic dispenser (Echo520 from Labcycte, San Jose, USA) equipped with Echo Dose Response software. Next, 6 μL of either (1) EGFR (0.5 nM, Thermo Fisher) and U*Light*-JAK-1 (Tyr1023) Peptide substrate (50 nM, PerkinElmer, TRF0121) mix or (2) ERBB4 (0.1 nM, Thermo Fisher) and U*Light*-poly GT peptide substrate (100 nM, PerkinElmer, TRF0100) mix was added. The reaction was started by addition of 2 μL ATP (final concentration 29 μM for EGFR assay; 0.59 μM for ERBB4 assay, Sigma-Aldrich) working solution and mixed using a Bioshake 5000 microplate shaker (Q Instruments, Jena, Germany). After 1 h incubation at room temperature, the reaction was stopped with 10 μL detection mix containing the 2 nM Europium-*anti*-phosphotyrosine (PT66) antibody (PerkinElmer, AD0068) and 10 mM EDTA. After a second incubation period of 1 h at room temperature, the FRET signal was measured at 340 nm excitation, 665 nm and 615 nm emission (for the U*Light*-substrate and Europium antibody, respectively) with an Envision microplate reader (PerkinElmer, Waltham, MA, USA) with 50 μs delay and 300 μs integration time. A kinase reaction without an inhibitor was set as positive control (using DMSO), representing the maximum readability of the system; conversely, a reaction without kinase was set as negative control using DMSO), representing the minimum readability of the system. Both positive and negative controls were run in parallel with every compound measurement as a calibration.

### Quantification and statistical analysis

#### Data analysis of barcode and luciferase reporter assays

For barcode reporter assays, sequencing reads of each transfected batch from each sample were normalized to their respective MLPmin sensor controls for every biological replicate and every condition. The MLPmin sensor-based normalization controlled technical aspects (cell number and transfection effects) and retained cell intrinsic effects (e.g., expression of signaling pathways). MLPmin sensors thus enabled to control for both target and cellular pathways activities, as observed in this study for e.g., the EGFR-dependent MAPK signaling captured by the EGR1p sensor (note that PC12 cells endogenously express EGFR, which, when activated, resulted in robust activation of MAPK/ERK signaling). Internal barcode replicates (3 for receptors, 2 for pathways; Figure 1A) were averaged and considered as one biological replicate. For luciferase assays, raw firefly values were used for analysis. Biological replicates were averaged, their respective standard errors were calculated, and both averages and standard errors were normalized to enable curve fitting from 0% to 100% for agonist dose-response curves. For antagonist treatments, the activity of the lowest compound concentration was set to 100%. Dose-response curves were visualized with R using the *drc* package. The robustness of the assays was calculated using the Z′ factor (cf. section on statistics). Z′ factors for barcoded assays obtained with strong inhibition of selected compounds are listed in Table S8. For heatmaps, the smallest concentration of each compound was set to 0 using the logarithmic scale to base 2. Heatmaps were plotted with R using the *ggplot2* package.

#### Data analysis of *in vitro* kinase assays

The mean values of the negative control reactions were subtracted from each reading of compound treated kinase reactions, followed by the division of the mean values of positive controls, from which the negative control means were also subtracted. Assays were run in triplicates. Dose-response curves and IC_50_ values were plotted and calculated using *drc* package in R.

#### Statistical analysis

Robustness of dose response assays was calculated using the Z′ factor that integrates both means and standard deviations of low and high concentration values.^101^ The Z′ factor was calculated by this formula: Z’ = 1-(3(SD_H_+SD_L_)/|mean_H_-mean_L_|). SD_H_ and SD_L_ are designated the standard deviation of the high and low control values, mean_H_ and mean_L_ are designated the averages of high and low control values. A value above Z ≥ 0.5 is considered as robust assay. Barcoded assays and *in vitro* kinase assays were run in triplicates, luciferase assays in six replicates, and Western blots in triplicates.

## Supplemental information

Document S1. Figures S1–S5 and Tables S2 and S8

Table S1. Plasmids used, related to Figure 1 Barcode sequences for RNA reporters linked to each sensor are also provided. n. a., not applicable.

Table S3. Relative IC50 values of dose response data from antagonist treatments, related to Figure 3 All data were obtained from barcoded ERBBprofiler assays. n. a., not applicable.

Table S4. Comparison of IC50 values obtained using the ERBBprofiler assay with those reported in the literature, related to Figure 3 Whenever available, a quantitative measure based on an IC_50_ value was retrieved. Otherwise, a reference to a qualitative measurement was provided. If no data from cellular assays (Western blot analyses, ELISA, proliferation assays) was available, data for biochemical assays were provided. Small effects are defined as antagonist actions that reduce the target/pathway activity to 70% of the full activity. n. a., not applicable.

Table S5. Combinations of plasmids used for each luciferase assay, related to STAR Methods n. a., not applicable.

Table S6. Combinations of plasmids used for each barcoded assay, related to STAR Methods n. a., not applicable.

Table S7. Oligos used for next-generation sequencing (NGS) and Tag&Pool purification, related to STAR Methods Sequences of Illumina sequencing primers for NGS and sequences of second-level barcode primers for the Tag&Pool purification method are listed.
