# Supplementary material for: Profiling of ERBB receptors and downstream pathways reveals selectivity and hidden properties of ERBB4 antagonists
Source: iScience. 2024 Jan 9;27(2):108839. doi: 10.1016/j.isci.2024.108839 (PMC10831936; doi:10.1016/j.isci.2024.108839)
Supplement: Document S1. Figures S1–S5 and Tables S2 and S8 [file mmc1.pdf]

iScience, Volume *27*

Supplemental information

Profiling of ERBB receptors and downstream pathways reveals selectivity and hidden properties of ERBB4 antagonists

Lukša Popović, Jan P. Wintgens, Yuxin Wu, Ben Brankatschk, Sascha Menninger, Carsten Degenhart, Niels Jensen, Sven P. Wichert, Bert Klebl, Moritz J. Rossner, and Michael C. Wehr

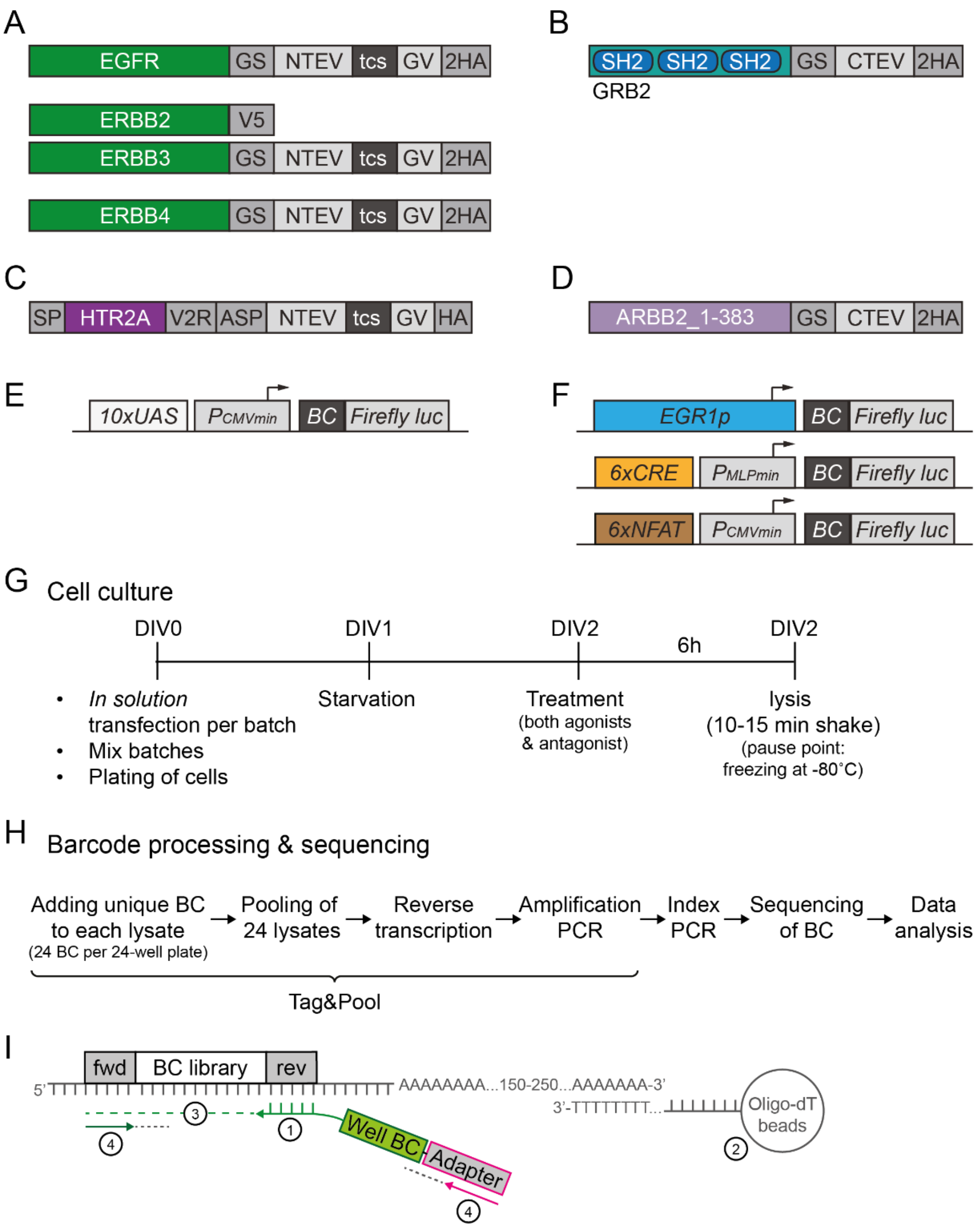

Figure S1. Schematic of the ERBBprofiler assay plasmids and experimental workflow, related to Figure 1 and related to STAR Methods.

(A) Schematic of plasmids encoding ERBB family receptors and serotonin receptor 2A (HTR2A). GS, glycin-serine linker; NTEV, N-terminal moiety of TEV protease; tcs, TEV protease cleavage site; GV, synthetic transcription factor GAL4-VP16; 2HA, double HA tag.

(B) Schematic of the plasmid encoding the RTK adapter 3xSH2(GRB2)-CTEV. CTEV, C-terminal moiety of TEV protease.

(C) Schematic of the plasmid encoding the serotonin receptor 2A (HTR2A). SP, signal peptide of the hemagglutinin virus; V2R, tail sequence of the vasopressin receptor 2A for enhanced beta-arrestin recruitment; ASP, semiflexible linker; HA, single HA tag.

(D) Schematic of the plasmid encoding the GPCR adapter ARBB2-CTEV comprising amino acid sequence 1-383 of ARBB2.

(E) Schmetic of the split TEV reporter plasmid. 10xUAS, 10x clustered upstream activating sequences; BC, barcode; CMVmin, minimal cytomegalovirus promoter; luc, luciferase.

(F) Schematics of pathway reporter plasmids for MAPK signaling (EGR1p, promoter of the EGR1 gene), cAMP and calcium signaling (6xCRE, 6x clustered cAMP responsive elements), and calcium signaling

(6xNFAT, 6x clustered response elements of the nuclear factor of activated T-cells). MLPmin, minimal major late promoter.
(G) Schematic representation of the experimental procedure for barcoded reporter assays in cell culture.
(H) Concept of barcode processing and sequencing. An amplification PCR followed by an index PCR using i5 and i7 indices for Illumina sequencing were conducted for collective library preparation for next-generation sequencing of barcodes. The first four steps comprise the Tag&Pool process.
(I) Scheme of the Tag&Pool technique to process mRNA transcripts containing barcodes. First, BC cassette-specific primers with a well BC (green) and an NGS adapter (grey/purple) were added into lysates of each well. This well BC primer annealed to the reverse (rev) region that was present in mRNA transcripts containing the BC library (step 1). Second, lysates were pooled and mRNAs from 24 different wells were purified with oligo-dT beads in one reaction (step 2). Third, cDNA was synthesized per pool using the well BC primer (step 3). Fourth, the first amplification PCR was conducted using a forward primer annealing to the forward (fwd) region and the well BC primer as reverse primer (step 4).

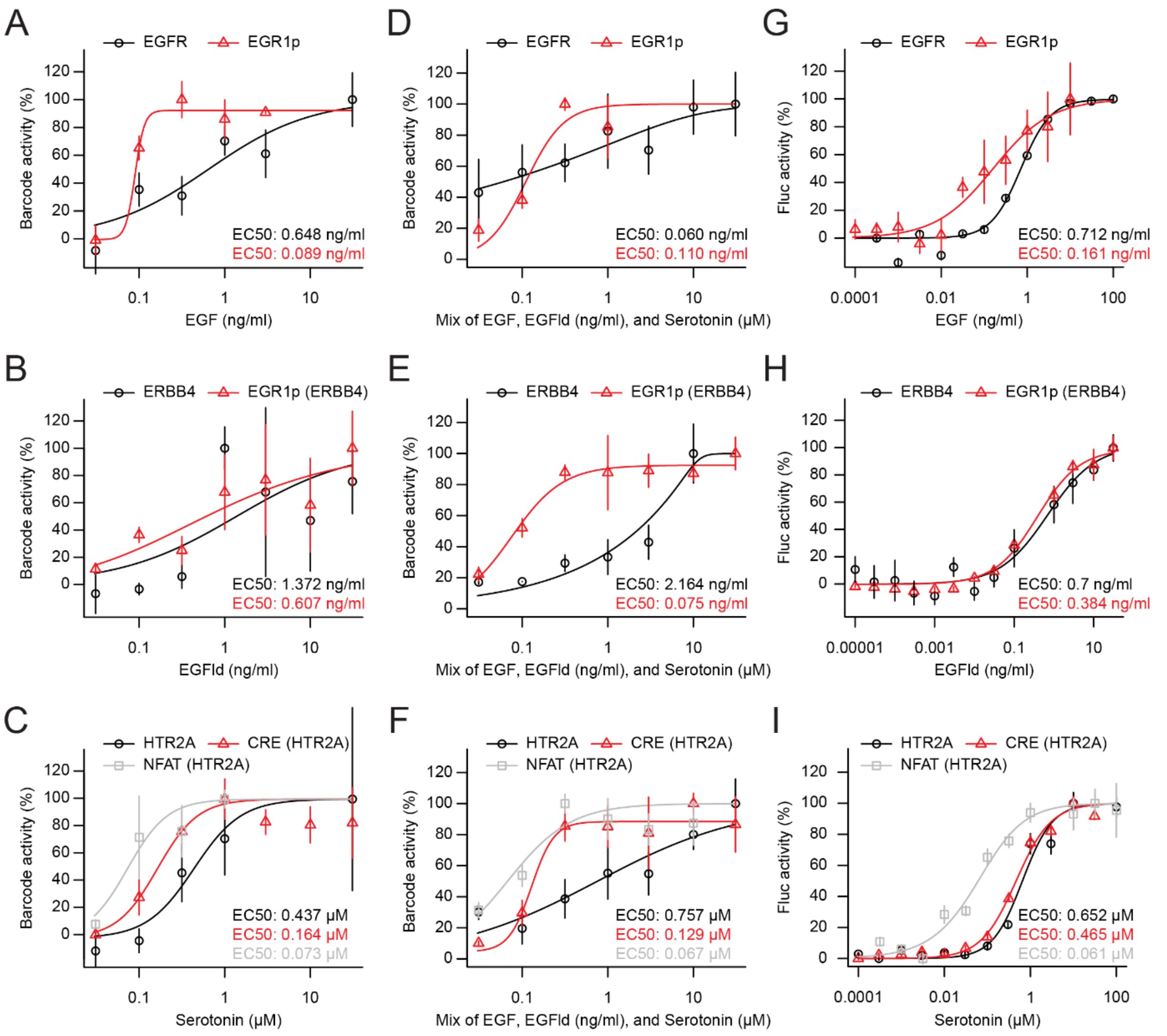

Figure S2. Receptor activation and downstream signaling by single ligands correlate with pooled treatments in barcoded and standard assays, related to Figure 2.

(A-F) Visualization of data that was extracted from Figure 2A, comparing barcoded receptor assays performed with split TEV and barcoded signaling assays performed with pathway sensors. Dose response graphs for EGFR and EGR1p (MAPK signaling) (A, D). ERBB4 and EGR1p (with ERBB4 co-transfected) (B, E), and HTR2A, CRE (with HTR2A co-transfected) (cAMP/calcium signaling), and NFAT (with HTR2A co-transfected) (calcium signaling) (C, F). Assays were either stimulated with single agonists (B-C) or a mix of agonists (D-F).

(G-I) Dose response luciferase assays for EGFR and EGR1p (G), ERBB4 and EGR1p (with ERBB4 co-transfected) (H), and HTR2A, CRE (with HTR2A co-transfected), and NFAT (with HTR2A co-transfected) (I) using single agonists. EGFld, EGF-like domain. Error bars represent SEM, n=3 for barcoded assays, and n=6 for luciferase assays.

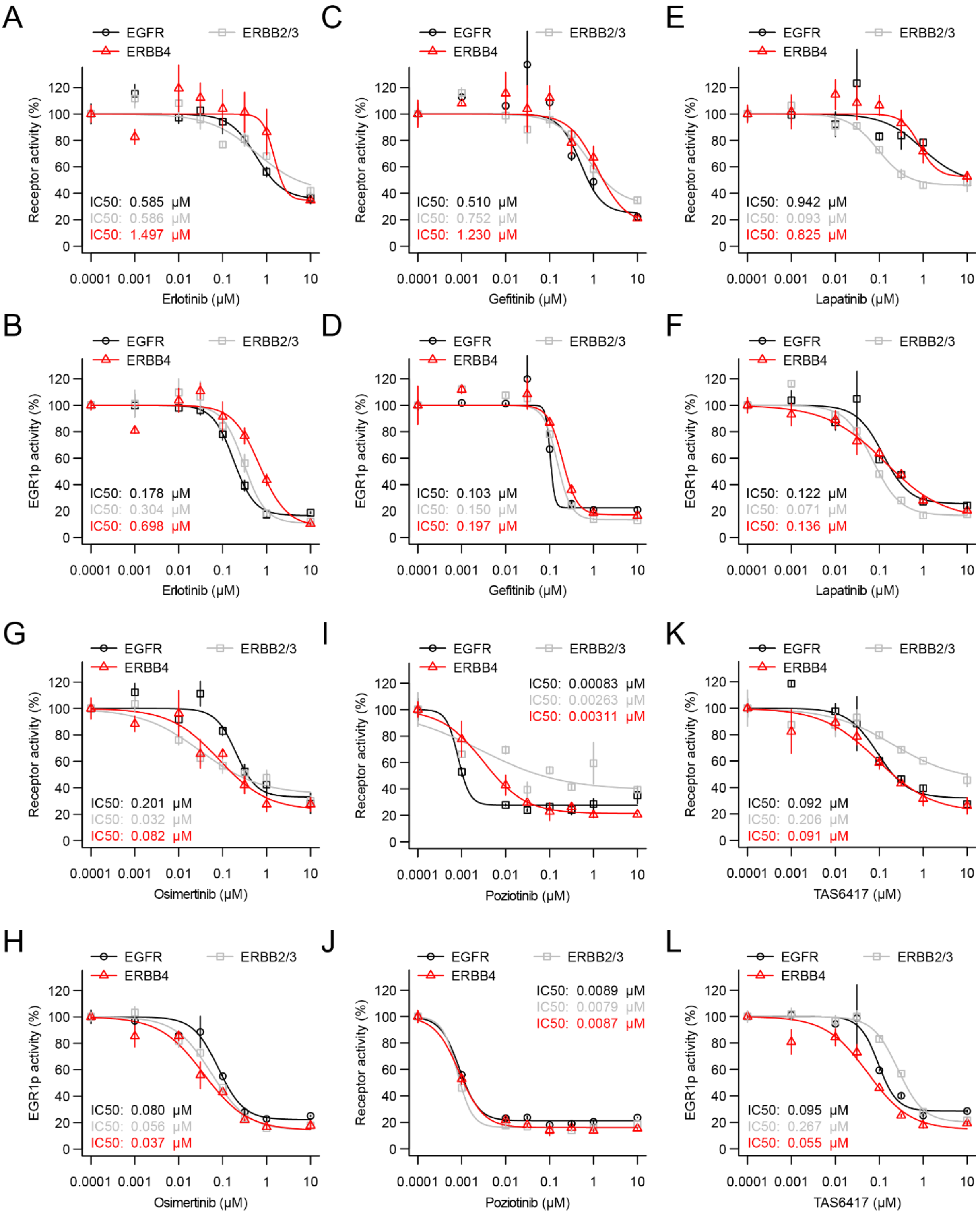

Figure S3. Novel antagonistic effects on ERBB receptors and pathways identified for osimertinib, poziotinib, and TAS6417, related to Figure 3.

(A-L) Dose response graphs showing effects of erlotinib (A, B), gefitinib (C, D), lapatinib (E, F), osimertinib (G, H), poziotinib (I, J), and TAS6417 (K, L). In addition to the increasing concentrations of the compounds shown, all assays contained constant concentrations of EGF (30 ng/ml), EGF-like domain (10 ng/ml), and serotonin (1 μM). Data was extracted from the heatmap shown in main Figure 3A. Assays for receptors were performed using barcoded split TEV assays, assays for MAPK signaling with barcoded EGR1p pathway sensors. Error bars represent SEM, n=3.

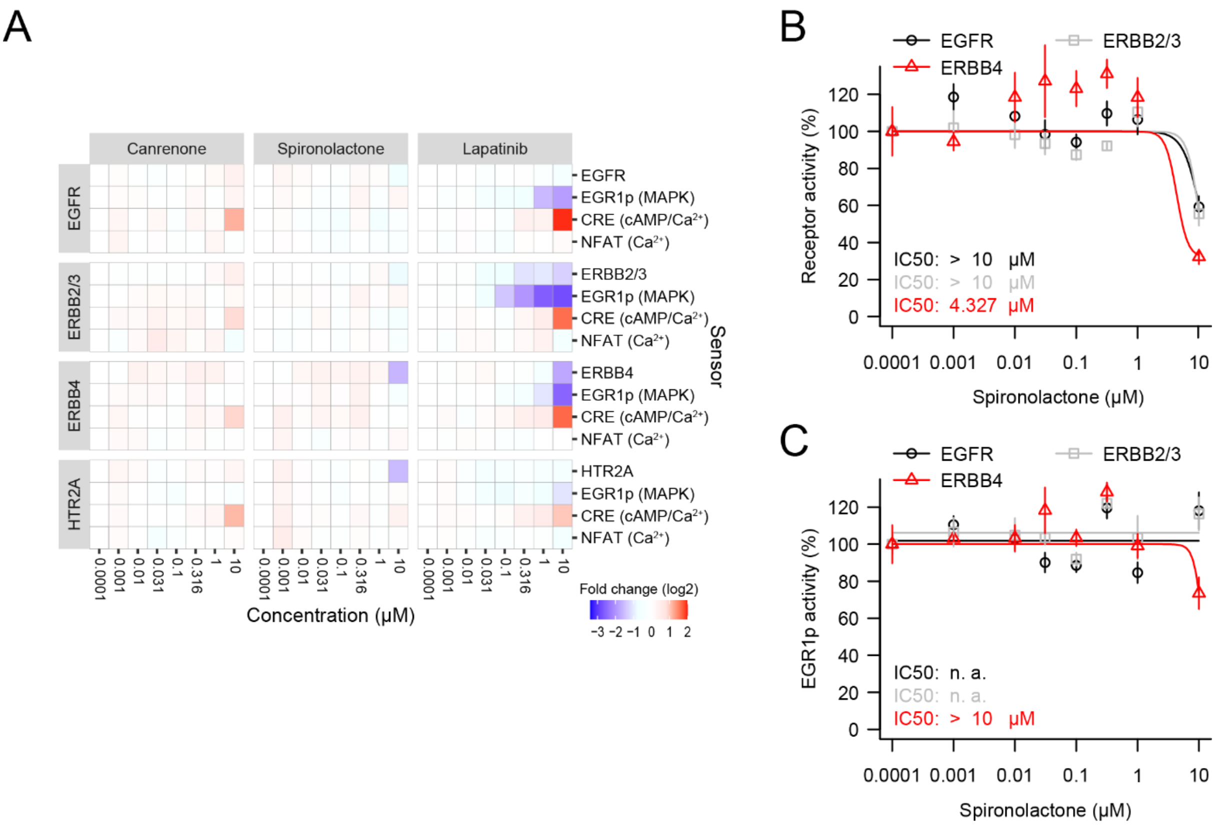

Figure S4. Spironolactone preferentially antagonizes ERBB4, related to Figure 5.
(A) Heatmap showing antagonistic effects of compounds spironolactone, canrenone, and lapatinib on ERBB receptors, HTR2A, and downstream signaling pathways. In addition to the increasing concentrations of the compounds shown, all assays contained constant concentrations of EGF (30 ng/ml), EGF-like domain (10 ng/ml), and serotonin (1 µM).
(B, C) Dose response graphs comparing drug selectivity for ERBB4 over EGFR and ERBB2/3 (B) and downstream MAPK signaling (C) of spironolactone. Data was extracted from the heatmap shown in (A). Assays for receptors were performed using barcoded split TEV assays, assays for MAPK signaling with barcoded EGR1p pathway sensors. Error bars represent SEM, n=3.

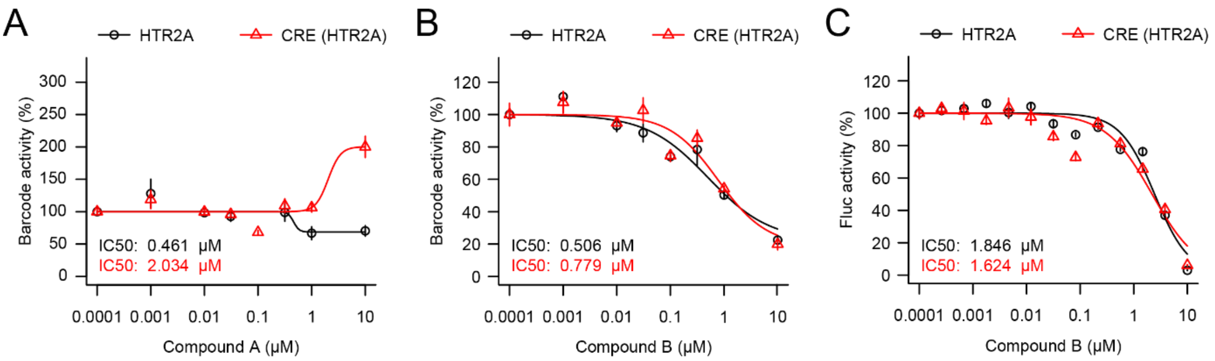

Figure S5. Compound B antagonizes HTR2A and downstream cAMP/Ca^{2+} signaling, related to Figure 5A.

(A) Dose response graph for HTR2A target (black) and CRE sensor pathway responses (red) of compound A. Data was extracted from the heatmap shown in main Figure 5A.

(B) Same as in (A), but for compound B.

(C) Dose response assays as in (B) using firefly luciferase as readout. In addition to the increasing concentrations of either compound A or compound B, all assays contained the constant stimulation mix as in main Figure 5A (EGF (30 ng/ml), EGF-like domain (10 ng/ml), and serotonin (1 μM)). Error bars represent SEM, n=3 for barcode assays (A, B), n=6 for luciferase assay (C).

Table S2. EC50 values of dose response data from agonist treatments performed, related to Figure 2. All data were obtained from a barcoded ERBBprofiler assay. n. a., not applicable.

Target/Pathway	EGF	EGFld	Serotonin	EGF/EGFld/Serotonin mix	Type of assay
EGFR	0.648 ng/ml	n.a.	n.a.	0.06 ng/ml	Target
ERBB2/3	n.a.	0.143 ng/ml	n.a.	0.85 ng/ml	Target
ERBB4	n.a.	1.372 ng/ml	n.a.	2.164 ng/ml	Target
EGR1p	0.089 ng/ml	n.a.	n.a.	0.11 ng/ml	Pathway
ERG1p (ERBB4)	0.093 ng/ml	0.607 ng/ml	n.a.	0.075 ng/ml	Pathway
HTR2A	n.a.	n.a.	0.437 µM	0.757 µM	Target
CRE	n.a.	n.a.	0.167 µM	0.198 µM	Pathway
CRE (HTR2A)	n.a.	n.a.	0.164 µM	0.129 µM	Pathway
NFAT	n.a.	n.a.	n.a.	n.a.	Pathway
NFAT (HTR2A)	n.a.	n.a.	0.073 µM	0.067 µM	Pathway

Table S8. Z’ factors for each receptor assay and corresponding pathway assay, related to STAR Methods. Values were determined from antagonist assays as indicated by the compound.

Assay	Z' factor	Compound
EGFR UAS (split TEV)	0.55	Gefitinib
EGFR EGR1p	0.69	Gefitinib
ERBB2/3 UAS (split TEV)	0.56	Lapatinib
ERBB2/3 EGR1p	0.90	Lapatinib
ERBB4 UAS (split TEV)	0.63	AG1478
ERBB4 EGR1p	0.83	AG1478
HTR2A UAS (split TEV)	0.54	Clozapine
HTR2A CRE	0.80	Clozapine
HTR2A NFAT	0.55	Clozapine
